# Establishment of an Immune-Related Gene Signature for Risk Stratification for Patients with Glioma

**DOI:** 10.1155/2021/2191709

**Published:** 2021-08-27

**Authors:** Jimin He, Chun Zeng, Yong Long

**Affiliations:** Department of Neurosurgery, Suining Central Hospital, Suining, China

## Abstract

Glioma is a frequently seen primary malignant intracranial tumor, characterized by poor prognosis. The study is aimed at constructing a prognostic model for risk stratification in patients suffering from glioma. Weighted gene coexpression network analysis (WGCNA), integrated transcriptome analysis, and combining immune-related genes (IRGs) were used to identify core differentially expressed IRGs (DE IRGs). Subsequently, univariate and multivariate Cox regression analyses were utilized to establish an immune-related risk score (IRRS) model for risk stratification for glioma patients. Furthermore, a nomogram was developed for predicting glioma patients' overall survival (OS). The turquoise module (cor = 0.67; *P* < 0.001) and its genes (*n* = 1092) were significantly pertinent to glioma progression. Ultimately, multivariate Cox regression analysis constructed an IRRS model based on *VEGFA*, *SOCS3*, *SPP1*, and *TGFB2* core DE IRGs, with a C-index of 0.811 (95% CI: 0.786-0.836). Then, Kaplan-Meier (KM) survival curves revealed that patients presenting high risk had a dismal outcome (*P* < 0.0001). Also, this IRRS model was found to be an independent prognostic indicator of gliomas' survival prediction, with HR of 1.89 (95% CI: 1.252-2.85) and 2.17 (95% CI: 1.493-3.14) in the Cancer Genome Atlas (TCGA) and Chinese Glioma Genome Atlas (CGGA) datasets, respectively. We established the IRRS prognostic model, capable of effectively stratifying glioma population, convenient for decision-making in clinical practice.

## 1. Introduction

Glioma is a common primary malignant brain tumor [1], with the average incidence of approximately six cases per 100,000 people in the world [2]. According to the WHO classification, glioma can be divided into four grades, including grades I, II, III, and IV, where grades I and II are low-grade glioma (LGG), including astrocytoma, oligodendroglioma, and mixed oligodendrogliomas, whereas grades III and IV are high-grade gliomas (HGG), such as glioblastoma (GBM), anaplastic astrocytoma, and anaplastic oligodendroglioma [3]. However, as time goes by, LGG will progress to invasive HGG [4–6]. On the basis of the statistical data of the Chinese Glioma Genome Atlas (CGGA), the overall survival (OS) of LGG is 78.1 months, with a 5-year survival rate of 67% [7] . Nevertheless, once patients progress to HGG, their OS will shorten to 14.4 months and the 5-year survival rate is roughly 9%. Even though they adopt positive treatments, such as surgery and chemotherapy, as well as radiotherapy, their 2-year survival rate is merely 43% [8].

Immunotherapeutic strategies in glioma arouse unprecedented attention in the public, where long-term tumor remission can be achieved with minimal side-effects [9]. The immune therapeutic methods included immune checkpoint inhibitors (ICI), peptide vaccines, dendritic cell vaccines, chimeric antigen receptor T cells, and oncolytic viruses [10]. The ICIs contain the programmed cell death protein (PD-1) and its ligand (PD-L1), considered as the main factors hindering immune response [11]. However, due to low and variable levels of PD-L1 in glioma cells [12, 13], most patients showed no significant increase in OS for anti-PD-1 therapy [14]. Thus, it is imperative to develop some potential consistently expressed immune-related biomarkers of the glioma. In recent years, some researchers propose that immune-related gene (IRG) signatures are involved in tumor prognosis, including pancreatic ductal adenocarcinoma [15], lung adenocarcinoma [16], neuroblastoma [17], and head and neck squamous cell carcinoma [18], as well as LGG [19]. In a previous study, Wang et al. have identified an immune-related lncRNA signature via construction of immune-lncRNAs and an immune-gene coexpression network, for predicting its prognostic value in the anaplastic gliomas [20]. Moreover, another previous study has established an immune-related risk signature for GBM, which can independently distinguish high-risk patients, and they also elucidated the relationship between local immune response and prognosis in GBM [21]. In addition, Zhang et al. constructed an IRG signature for risk stratification and developed a prognostic nomogram for survival prediction in LGG patients [19]. However, most researches established prognostic models based on IRG signatures [19, 21–23], but they have yet to illuminate whether progression-related differentially expressed IRGs (DE IRGs) can effectively perform risk stratification for patients subjected to glioma.

Hence, this present study was undertaken to develop a prognostic model via screening progression-related core DE IRGs [24], for stratifying glioma patients by risk score and providing potential immunotherapeutic targets for inhibition of glioma progression and improvement of patients' prognosis.

## 2. Materials and Methods

### 2.1. Data Downloading and Processing

By searching the GEO database (http://www.ncbi.nlm.nih.gov/geo/), original data (CEL format) of GSE4290 were downloaded and decompressed to perform weighted gene coexpression network analysis (WGCNA). The LGG and HGG samples from GSE45921 [25], GSE15824 [26], GSE43378 [27], and GSE4290 [28] datasets were also selected for differential expression analysis, all of which have same annotation platform, namely, GPL570 (HG-U133_Plus_2, Affymetrix Human Genome U133 Plus 2.0 Array), with 54,675 probes. Besides, transcriptome and clinical data (including sample id, age, gender, status, survival time, tumor grade, isocitrate dehydrogenase (*IDH*) status, and O-6-methylguanine-DNA methyltransferase (*MGMT*) status) of all glioma samples from the TCGA-LGG (level 3) and TCGA-GBM (level 3) were downloaded from the TCGA database (https://portal.gdc.cancer.gov/) and cBioPortal for Cancer Genomics database (http://www.cbioportal.org/), respectively. Next, we attained a total of 2498 IRGs from the Immunology Database and Analysis Portal (ImmPort) (https://www.immport.org/shared/home) [18, 29]. Subsequently, we collected RNA-seq data and corresponding clinical information from the CGGA database (http://www.cgga.org.cn/) [30, 31] for further verification.

### 2.2. Weighted Gene Coexpression Network Analysis

Genes with top-ranking 5000 expression data of the GSE4290 dataset were used to implement WGCNA. Then, coexpression modules were constructed using the “WGCNA” package in the RStudio software (Version 3.5.0). To eliminate outliers, the parameters of height and minsize were set to 110 and 30, respectively. Using the power function *a*_mn_ = |*c*_mn_|^*β*^ (*a*_mn_: adjacency between gene m and gene n, *c*_mn_: Pearson correlation, *β*: the soft threshold), a weighted adjacency matrix was constructed [32, 33]. Then, the topological overlap matrix (TOM) was converted to the adjacency matrix to measure the network connectivity of these genes [32]. Subsequently, genes with absolute high correlation were classified into gene modules according to the TOM-based dissimilarity metric [33]. In addition, coexpression modules were uncovered using the blockwiseModules function of the “WGCNA” package [34, 35]. Through establishment of a hierarchical clustering tree diagram of selected gene expression values and analysis of adaptive branch cutting [36], functional modules were visualized with different colors [37], where genes without being enriched in any module were assigned to the gray module [38]. Module eigengene (ME) is defined as the main component of the first standardized expression profile [39] and also is thought as the representative gene expression profile in modules [33, 40]. Furthermore, module significance tends to be highly correlated with the correlations between ME and clinical traits [41]. And gene significance (GS) is considered to be the absolute values of the correlation coefficients between genes and traits, and module membership (MM) is defined as the correlations between the eigengene modules and gene expression profile [34, 42]. Accordingly, a preliminary correlation between modules and clinical traits was revealed via the WGCNA method.

### 2.3. Identification of Differentially Expressed Genes (DEGs) and Their IRGs (DE IRGs)

The original data from four GEO datasets (including GSE45921, GSE15824, GSE43378 and GSE4290) were decompressed and implemented normalization, background correction, and log2 transformation via the “affy” package [43, 44]. And missing values were filled using the impute.knn function [45]. Additionally, the ComBat algorithm in the “sva” package was adopted for removing batch-batch difference [46]. Subsequently, differential expression analysis between HGG and LGG samples was performed using the “limma” package. If multiple probes matched to the same gene symbol, the median expression values were selected as the expression levels of these genes. Besides, cutoff values of DEGs were set to ∣log_2_FoldChange (log_2_FC) | ≥1.0 and adjusted *P* < 0.05. To further discover glioma progression-related DE IRGs, a Venn diagram was used based on an overlapping region of DEGs and IRGs, as well as genes in the significant progression-related modules.

### 2.4. Functional Enrichment Analyses for DE IRGs

The Gene Ontology (GO) term and Kyoto Encyclopedia of Genes and Genomes (KEGG) functional enrichment analyses were performed for DE IRGs via the “ClusterProfiler” [47] package. GO term enrichment analysis contained three categories, namely, molecular function (MF), cellular component (CC), and biological processes (BP) [48]. Additionally, KEGG (http://www.genome.jp/kegg/) is a bioinformatics resource that links genome or molecular datasets to networks [49]. The *P* value less than 0.05 was regarded as the threshold.

### 2.5. Construction of a PPI Network and Identification of Clusters

A protein-protein interaction (PPI) network of DE IRGs was constructed using the STRING (https://string-db.org/) [50] website via setting the minimum required interaction score to 0.900 and the max number of interactors to show to no more than 50, and visualized in the Cytoscape software [51]. Furthermore, the median degree was calculated by the CentiScaPe 2.0 plug-in, a tool for determining the most significant nodes [52]. And the molecular complex detection (MCODE) [53] plug-in was capable of visualizing the highly connected clusters of the PPI network. Thus, significant nodes were selected as core genes for the next analysis. A GOChord function in the “GOplot” package was employed to display the relationships that genes were coupled with GO terms through ribbons [54].

### 2.6. Survival Analysis

To disclose the predictive value of expression levels of DE IRGs for glioma survival, we adopted a log rank test to perform Kaplan-Meier (KM) survival analyses.

### 2.7. Establishment of an Immune-Related Risk Score (IRRS) Model

We screened out the most significant nodes via the previous methods. The TCGA samples were used to construct and assess the IRRS model. Firstly, univariate Cox regression analysis was performed to screen those genes with *P* < 0.05. Subsequently, the IRRS model was established by multivariate Cox regression analysis based on the Akaike Information Criterion (AIC) algorithm [55]. The risk score formula was erected via the coefficients (*β*) of each gene from the multivariate Cox regression analysis and their expression levels. The calculation formula of the risk score was as follows: risk score = expression level of mRNA1 × *β*1_mRNA1_ + expression level of mRNA2 × *β*2_mRNA2_ + ⋯+expression level of mRNAn × *βn*_mRNAn_. Then, the time-dependent receiver operating characteristic (ROC) curves for predicting the 1-year, 3-year, and 5-year OS showed the predictive performance of this IRRS model via the “survivalROC” package [56]. In addition, the concordance index (C-index), ranging from 0.5 to 1.0, is an indicator for evaluating this model's predictive ability. The closer to 1.0 the C-index, the better the discriminatory ability of the IRRS model [57]. Next, considering that 5 years is a key time point for evaluating the prognosis of glioma patients, we have stratified patients into high- and low-risk groups via adopting the optimal cutoff value of the risk score at the 5-year time point in ROC curves [58]. Subsequently, survival analysis was implemented using the “survival” and “survminer” packages via log rank test, and the 5-year survival rate also was acquired.

### 2.8. Evaluation of the Independent Prognostic Value of the IRRS Model

We have performed univariate and multivariate Cox regression analyses for the IRRS model and clinical characteristics, that is, a combination of age (<60 and ≥60), gender (male and female), tumor grade (low grade and high grade), risk score (low risk and high risk), *IDH* status (mutant and wild-type), and *MGMT* status (methylated and unmethylated). Subsequently, the nomogram with 1-year, 3-year, and 5-year OS rates was developed for clinical use [59]. Moreover, calibration curves were utilized to evaluate the agreement of predictive and observed probabilities of 1-year, 3-year, and 5-year OS from the nomogram.

### 2.9. Statistical Analysis

Statistical analysis was conducted in the RStudio software (Version 3.5.0) and GraphPad Prism software (Version 5.0). Survival curves were established via the KM method and log-rank test. Calculation of the area under ROC curves of 1-year, 3-year, and 5-year survival rates was implemented based on the “survivalROC” package [56]. The “rms” package was applied to perform nomogram analysis. *P* < 0.05 denoted the presence of statistically significant difference.

## 3. Results

### 3.1. Data Processing

**Figure 1:**
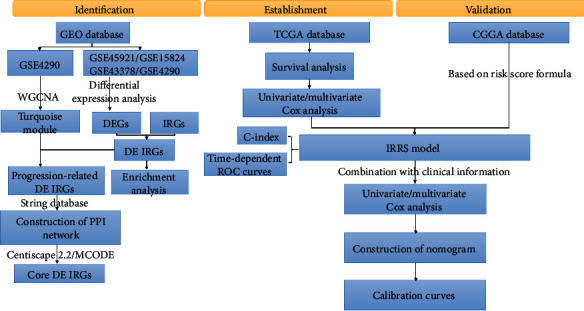
The flow chart of the present study.

The flowchart of the present study design is presented in Figure 1. First, we selected the top-ranking 5000 gene expression values of the GSE4290 dataset based on the median absolute derivation (MAD) algorithm for performing WGCNA. Second, integrated transcriptome analysis was conducted in a total of 251 samples, including 179 HGG and 72 LGG samples, derived from four GEO datasets, among which 7 HGG and 15 LGG in the GSE45921 dataset, 19 HGG and 7 LGG in the GSE15824 dataset, 45 HGG and 5 LGG in the GSE43378 dataset, and 108 HGG and 45 LGG in the GSE4290 dataset. In addition, 687 duplicate IRGs were removed from IRG group. Then, fragments per kilobase per million normalized expressions of RNA-seq data (level 3) of 529 LGG and 169 GBM samples in the TCGA database were used for construction of the prognostic model. Due to 10 samples lacking information of survival time and status, we ultimately used 688 glioma samples to construct the prognostic model and validate its predictive performance. Additionally, 422 primary glioma patients from the CGGA database were selected as the external validation set.

### 3.2. Construction of a Weight Gene Coexpression Network and Identification of Grade Progression-Related Modules

**Figure 2:**
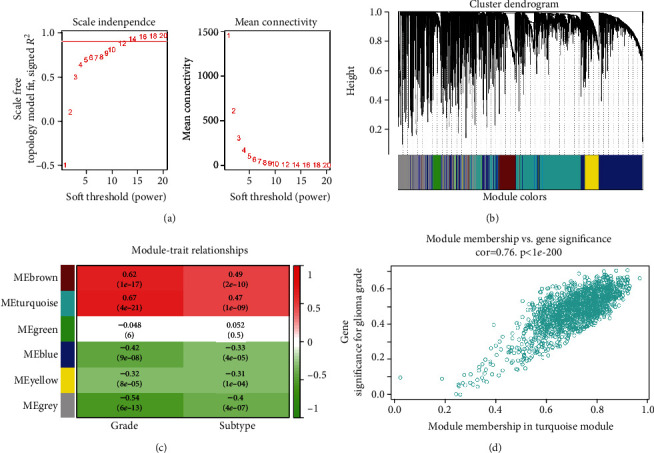
Weighted gene coexpression network analysis. (a) Identification of the soft threshold power on the basis of the criteria of the scale-free network. The left panel shows the relationship between the scale-free fit index (*y*-axis) and soft threshold power (*x*-axis). And the right panel shows the impact of soft threshold power (*x*-axis) on the mean connectivity (degree, *y*-axis). (b) Cluster dendrogram of gene coexpressed modules. A gene clustering dendrogram is plotted using the dissimilarity measure (1-TOM). The colored horizontal bars below the clustering dendrogram represent the modules labeled with different colors, where the gray module shows no genes enriched in any module. (c) The heat map of the correlations between module eigengenes and clinical features of glioma. Each column represents a clinical trait (including grade and subtype) and each row represents a module. Each cell contains the corresponding correlation coefficient on the first row and *P* value on the second row. The red and green colors show the positive and negative correlations, respectively. And the darker the color of the cell, the stronger the correlation between modules and clinical traits. (d) Scatter plot of the correlation between gene significance for the glioma grade and module membership in the turquoise module (cor = 0.76; *P* < 0.0001). TOM: topological overlap matrix.

First, we used a hierarchical clustering method to detect the outliers, enabling two samples to be eliminated (“GSM97895” and “GSM97932”). The soft threshold value was equal to 12 via the pickSoftThreshold function, shown in Figure 2(a). Second, we employed the blockwiseModules function of the “WGCNA” package to acquire six modules, including brown, turquoise, green, blue, yellow and gray modules, with genes ranging from 149 to 1552, where the gray module represented genes without being enriched, which is visualized in Figure 2(b). Third, we analyzed the relationship between samples and clinical traits (such as grade and subtype of glioma) and obtained the correlation of modules with traits. And Figure 2(c) showed that the turquoise module was significantly positively associated with glioma grade (cor = 0.67, *P* < 0.0001). Then, a scatter plot of MM vs. GS in the turquoise module was drawn, with a correlation coefficient of 0.76 (*P* < 0.0001) (Figure 2(d)). Additionally, 1092 genes in the turquoise module were identified (Supplementary Table 1).

### 3.3. Differentially Expressed Genes (DEGs) and Their IRGs (DE IRGs), as well as Functional Enrichment Analyses

**Figure 3:**
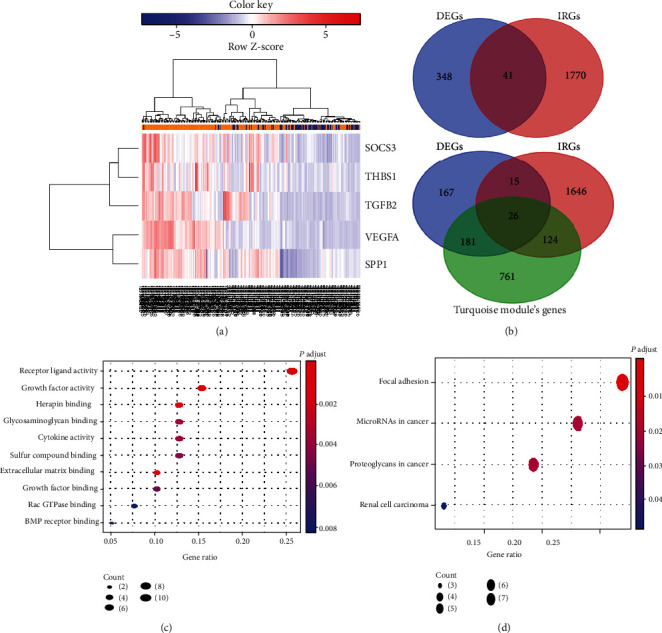
Identification of DE IRGs and functional enrichment analyses. (a) The clustering heat map of five DE IRGs. The row and column represent genes and samples, respectively. (b) Venn plots reveal 41 DE IRGs and 26 progression-related DE IRGs. (c) GO term enrichment analysis for DE IRGs. (d) KEGG pathway enrichment analysis for DE IRGs. DEGs: differentially expressed genes; IRGs: immune-related genes; DE IRGs: differentially expressed immune-related genes; *SOCS3*: suppressor of cytokine signaling 3; *THBS1*: thrombospondin 1; *TGFB2*: transforming growth factor beta 2; *VEGFA*: vascular endothelial growth factor A; *SPP1*: secreted phosphoprotein 1; GO: Gene Ontology; KEGG: Kyoto Encyclopedia of Genes and Genomes.

Differential expression analysis between HGG (*n* = 179) and LGG (*n* = 72) samples was completed via the “limma” package. According to the presetting cutoff value, 389 DEGs (Supplementary Table 2) were obtained for further analysis, with upregulation of 213 and downregulation of 176 DEGs.  Figure 3(a) shows a clustering heat map that reveals the expression levels of five core DE IRGs. We selected the 41 overlapping genes in the DEG and IRG groups via Venn plot as DE IRGs. Thus, as shown in Figure 3(b), 26 progression-related DE IRGs located in the intersection region among the DEG, IRG, and turquoise module's gene groups. Subsequently, Figures 3(c) and 3(d) present the results of GO term and KEGG pathway enrichment analyses, respectively, which clarified that these DE IRGs were mainly enriched in receptor ligand activity, growth factor activity, extracellular matrix binding, etc. (GO term), as well as focal adhesion (KEGG pathway).

### 3.4. Construction of a PPI Network and Identification of Clusters

**Figure 4:**
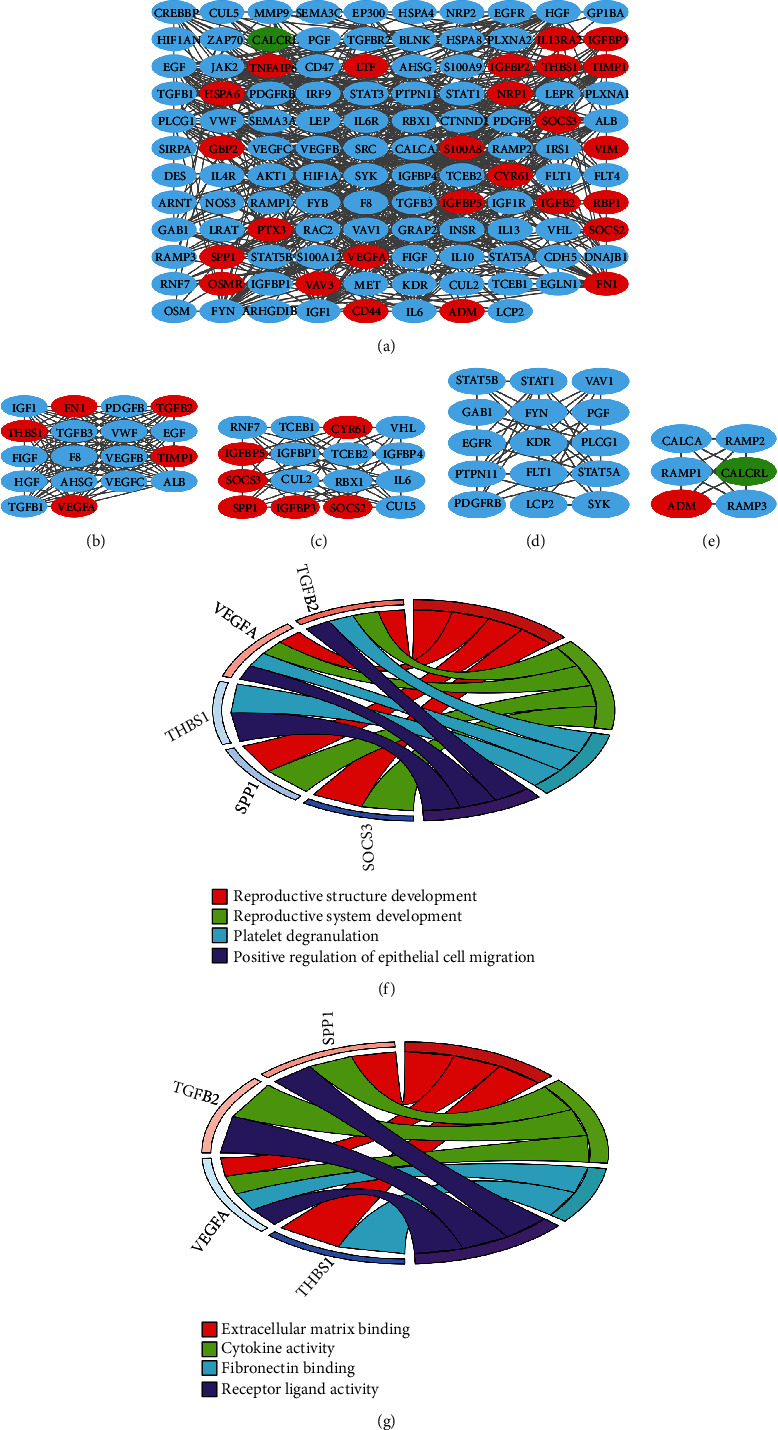
Construction of a PPI network, identification of clusters, and GOChord plot for core DE IRGs. (a) PPI network with 118 nodes and 705 edges, with the red and green representations of upregulated and downregulated DEGs, respectively. (b) Cluster A, (c) cluster B, (d) cluster C, and (e) cluster D. GOChord plot for five core DE IRGs in the (f) “BP” category and (g) “MF” category. PPI: protein-protein interaction; DEGs: differentially expressed genes; DE IRGs: differentially expressed immune-related genes; BP: biological process; MF: molecular function.

A PPI network of 26 progression-related DE IRGs was constructed by the STRING tool and was visualized via the Cytoscape software, with 118 nodes and 705 edges (Figure 4(a)). The median degree, as the threshold of PPI network, was 11.94 calculated by the CentiScaPe 2.2 plug-in. Subsequently, a total of four closely connected clusters were discovered (Table 1), with a score more than 6.0 using the MCODE algorithm, where the parameters of degree cutoff, node score cutoff, *k*-score, and max. depth were set to 2.0, 0.2, 2.0, and 100, respectively (Figures 4(b)–4(e)). Ultimately, we have identified five core DE IRGs (including *VEGFA*, *SOCS3*, *THBS1*, *SPP1*, and *TGFB2* genes) with a degree more than 12 in clusters A and B, which met the requirements mentioned above. Additionally, GOChord diagrams disclosed that most of core DE IRGs were enriched in the positive regulation of epithelial cell migration (BP, Figure 4(f)) and extracellular matrix binding and cytokine activity, as well as receptor ligand activity (MF, Figure 4(g)).

### 3.5. Evaluation of the Impact of Core DE IRGs on Glioma Prognosis

**Figure 5:**
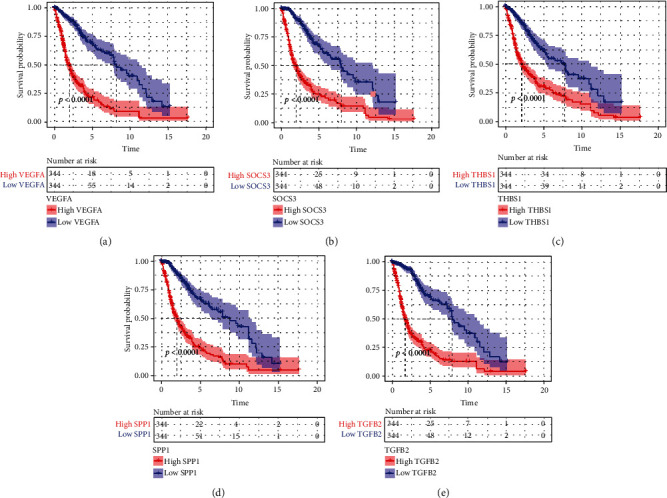
Survival curves uncover the relationships between five core DE IRGs and glioma patients' survival in the TCGA cohort: (a) *VEGFA* (HR = 4.03, 95% CI: 3.16-5.15, *P* < 0.0001), (b) *SOCS3* (HR = 4.26, 95% CI: 3.35-5.43, *P* < 0.0001), (c) *THBS1* (HR = 2.93, 95% CI: 2.31-3.72, *P* < 0.0001), (d) *SPP1* (HR = 3.80, 95% CI: 2.98-4.85, *P* < 0.0001), and (e) *TGFB2* (HR = 4.64, 95% CI: 3.64-5.92, *P* < 0.0001). DE IRGs: differentially expressed immune-related genes; TCGA: The Cancer Genome Atlas; *VEGFA*: vascular endothelial growth factor A; *SOCS3*: suppressor of cytokine signaling 3; *THBS1*: thrombospondin 1; *SPP1*: secreted phosphoprotein 1; *TGFB2*: transforming growth factor beta 2.

Survival curves of five core DE IRGs indicated that high levels of core DE IRGs were associated with shorter OS of glioma patients (Table 2, Figure 5, *P* < 0.0001). Additionally, the 5-year survival rates of patients with overexpressed *VEGFA*, *SOCS3*, *THBS1*, *SPP1*, and *TGFB2* genes in KM survival curves separately were 20.38%, 23.85%, 29.61%, 24.27%, and 21.93% based on the TCGA cohort, whereas in the CGGA dataset, for patients with high expression levels of *VEGFA*, *SOCS3*, *THBS1*, *SPP1*, and *TGFB2* genes, the 5-year survival rates were 33.70%, 30.20%, 36.70%, 38.30%, and 37.60%, respectively (Table 2).

### 3.6. Establishment of the IRRS Model

**Figure 6:**
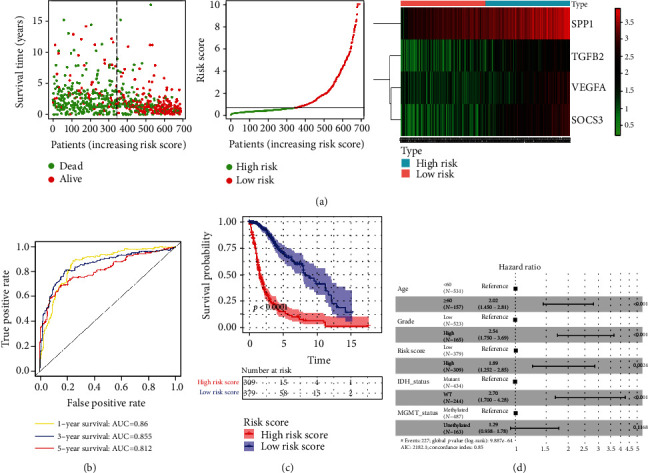
Establishment of an IRRS model in the TCGA cohort. (a) The distribution of risk score in glioma patients based on the IRRS model and survival status of each glioma patient, as well as a heat map of four DE IRG expressions in high- and low-risk groups. (b) ROC curves at 1-year (AUC: 0.86), 3-year (AUC: 0.855), and 5-year (AUC: 0.812) time points for predicting its prognostic performance in patients with glioma. (c) Survival curve reveals high-risk score patients associated with unfavorable outcome, with a 5-year survival rate of roughly 14.3%. (d) Multivariate Cox regression analysis discovers the potentiality of this IRRS model as an independent prognostic factor (HR = 1.89, 95% CI: 1.252-2.85, *P* = 0.0024). IRRS: immune-related risk score; TCGA: The Cancer Genome Atlas; DE IRGs: differentially expressed immune-related genes; AUC: area under the curve; OS: overall survival; ROC: receiver operating characteristic.

These core DE IRGs were used to perform univariate Cox regression analysis, screening genes with a *P* value less than 0.05, which were included for conducting multivariate Cox regression analysis via the AIC algorithm (the smallest value of 2753.8) for establishment of a prognostic model. Finally, we constructed an IRRS model composed of four DE IRGs via the following formula: risk score = 0.20365 × *VEGFA* + 0.12448 × *SOCS*3 + 0.26231 × *SPP*1 + 0.12930 × *TGFB*2. And C-index was 0.811 (95% CI: 0.786-0.836) for the IRRS model, showing the superiority of this model for predicting the probability of OS in glioma population (Table 3). Subsequently, we used diagrams of survival status' distribution and risk score of each glioma patient, as well as four gene expression heat maps to illuminate the IRRS model's role in glioma patients (Figure 6(a)). Further evidence implied that the areas under ROC curves for evaluating 1-year, 3-year, and 5-year OS were 0.86, 0.855, and 0.812, respectively (Figure 6(b)). The optimal cutoff value of the risk score for forecasting the 5-year survival in ROC curves was 0.87894, which was used to classify glioma patients into high- and low-risk populations and then generate a survival curve, revealing that the 5-year survival rate was merely 14.30% in the high-risk group (Figure 6(c)). As is shown in Table 4, the correlations between the risk score and clinicopathological characteristics were described. Also, univariate and multivariate Cox regression analyses based on the risk score and clinical characteristics indicated that this IRRS model was able to be an independent prognostic factor (Figure 6(d)), with an HR value of 1.89 (95% CI: 1.252-2.85, *P* = 0.0024) (Table 5). Due to the trait of gender showing no statistical difference in univariate Cox regression analysis (HR = 1.26, 95% CI: 0.986-1.61, *P* = 0.0645), we excluded the factor of gender; then, we included the rest of the indicators to perform multivariate Cox regression analysis. Furthermore, gliomas' grade was found to be a risk factor of glioma patients (HR = 2.54, 95% CI: 1.750-3.69, *P* < 0.001). Also, *IDH* wild-type also was characterized by an independent prognostic value for glioma patients (HR = 2.70, 95% CI: 1.700-4.28, *P* < 0.001).

### 3.7. Validation of the IRRS Model

**Figure 7:**
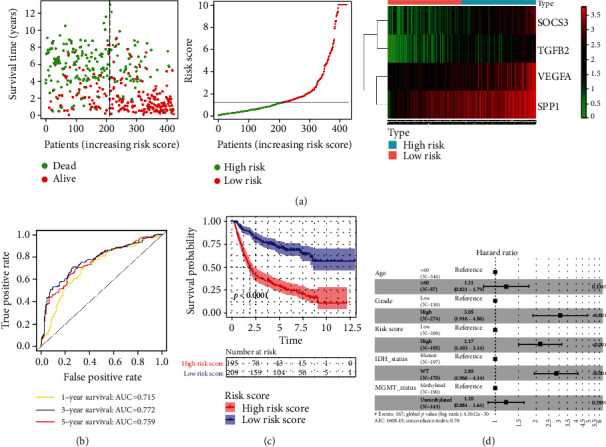
Validation of the IRRS model in the CGGA dataset. (a) The distribution of risk score, survival status of every patient with glioma, and heat map of four DE IRGs. (b) ROC curves of this IRRS prognostic model for evaluating the predictive probability for gliomas' 1-year (AUC: 0.715), 3-year (AUC: 0.772), and 5-year (AUC: 0.759) OS. (c) KM curve indicates glioma patients with high risk has dismal prognosis, with 5-year survival rate of nearly 29.8%. (d) Multivariate Cox regression analysis for the risk score and clinical characteristics denotes a superior prognostic value of the IRRS model for glioma patients (HR = 2.17, 95% CI: 1.493-3.14, *P* < 0.0001). IRRS: immune-related risk score; CGGA: Chinese Glioma Genome Atlas; DE IRGs: differentially expressed immune-related genes; AUC: area under the curve; OS: overall survival; KM: Kaplan-Meier; ROC: receiver operating characteristic.

The CGGA cohort was selected as an external validation set. The survival status of each patient, their risk scores' distribution, and the heat map of four genes are shown in Figure 7(a). Similarly, the areas under the curve (AUC) of the risk score at 1-year, 3-year, and 5-year time points of ROC curves were 0.715, 0.772, and 0.759, respectively (Figure 7(b)). Also, the optimal cutoff value of 1.27, calculated by the “survivalROC” package, divided patients into high- and low-risk groups; then, the survival curve elucidated that the 5-year survival rate was merely 29.80% in high-risk glioma patients (Figure 7(c)). Meanwhile, the IRRS model was demonstrated that it can become an independent prognostic indicator (HR: 2.17, 95% CI: 1.493-3.14, *P* < 0.0001, Figure 7(d)).

### 3.8. Development of a Nomogram

**Figure 8:**
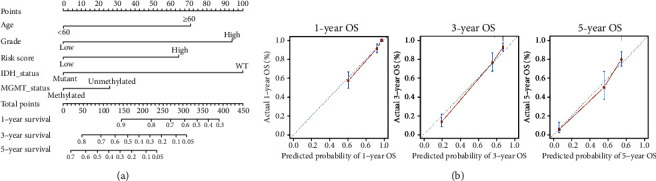
(a) Development of a nomogram and (b) calibration curve analysis. The *x*-axis and *y*-axis separately represent the predicted and actual OS from the nomogram. The diagonal line indicates that the predicted probability is consistent with the actual probability. The solid line and vertical line show the predicted nomogram and 95% confidence interval, respectively. WT: wild type; OS: overall survival.

A nomogram was erected to predict 1-year, 3-year, and 5-year OS of glioma patients via combing the IRRS prognostic model and significant clinical characteristics (Figure 8(a)), which appeared to be conducive to clinical use. Furthermore, calibration curves substantiated a superior consistency between predicted and observed values at probabilities of 1-year, 3-year, and 5-year OS of the nomogram, as described in Figure 8(b).

### 3.9. Comparison with Other Prognostic Models of Glioma

Via searching related literature about glioma's prognostic models, we compared these models with this IRRS model (Table 6). Our model had moderate accuracy with AUC of 1 year, 3 years, and 5 years more than 0.70 both in the TCGA and CGGA databases. Despite the presence of higher accuracy in other models, their models were composed of more than ten genes, which restricted their widespread application.

## 4. Discussion

With the increasing significance of IRG signatures on tumors' prognosis [29, 77, 78], it is imperative to throw light on their prognostic value for tumors. In our present study, four progression-related DE IRG (including *VEGFA*, *SOCS3*, *SPP1*, and *TGFB2* genes)constituting a signature can perform risk stratification for glioma patients and disclosed that patients with high risk seemed to have an approximate 14.3%-29.8% 5-year survival rate. Besides, we speculated that these overexpressed core genes might participate in the dismal prognosis of patients suffering from glioma via the extracellular matrix (ECM) binding's molecular function. ECM functions as a pivotal role in communicating with a variety of cell types, such as fibroblasts, immune cells, endothelial cells, epithelial cells, and pericytes, via all sorts of cell surface receptors, for regulating their functions and behaviors [79].

*VEGFA*, called vascular endothelial growth factor A, belongs to the *PDGF/VEGF* growth factor family and plays an essential part in inducing neovascularization [80] and promoting endothelial cell proliferation and migration. For example, the enzyme for degrading ECM can release active fragments from the matrix substance and activate growth factors, including the heparin-binding epidermal growth factor, insulin-like growth factor, epidermal growth factor, *VEGF*, and fibroblast growth factor-2, not only promoting the growth but also propelling invasion and neovascularization of tumors [81, 82]. Also, *VEGFA* can act as a crucial regulator of the cancer-immune circulation via generating considerable modifications, inducing immune tolerance and causing tumor immune evasion. Furthermore, soluble proteins coded by the *VEGFA* gene were involved in the mechanism of inducing angiogenesis and immunosuppressive responses in gastric cancer [83]. In addition, Hatva et al. [84] measured the expression of *VEGF* in both the normal brain vessel system and glioma cells; then, they concluded that *VEGF* was manifestly overexpressed in malignant glioma cells and their corresponding receptors were induced, which played a cardinal role in the angiogenesis of tumors. Besides, Zhang et al. [85] found that escalation of *VEGFA* participated in the progression of glioma and lowered patients' OS. Additionally, a high level of *VEGFA* was relevant to hypoxia, angiogenesis, and immune suppression of the tumor microenvironment (TME) [86], which elaborated the mechanism of tumors' innate resistance to ICIs and uncovered that upregulated *VEGFA* enhanced the malignancy of tumor cells [87]. Some evidence indicated that a high level of *VEGFA* had the potentiality of facilitating treatment efficacy based on target therapy. Compared to monotherapy, in the bearing-tumor mice without high expression of *VEGFA*, combination treatment showed no synergistic effect in antitumor efficacy. Surprisingly, in treatment of tumors with overexpressed *VEGFA*, antiangiogenesis therapies have transformed the immunosuppressive TME and enhanced the effects of ICIs [87]. Thus, *VEGFA* can serve as not only a prognostic biomarker for predicting gliomas' prognosis but also a potential immunotherapeutic target in the near future.

*SOCS3*, suppressor of cytokine signaling 3, is one of the most potent members of the *SOCS* family and encodes a group of *STAT* inhibitors induced by *STAT*, which contains a motif of the kinase-inhibitory region (KIR) and directly inhibits signal transduction via suppressing the Janus kinase (JAK) catalytic activity [88, 89]. To our knowledge, suppressive *SOCS3* is a negative modulator of cytokine signaling via directly inhibiting JAKs, functioning as a key regulator of the immune system [90]. Besides, in the central nervous system, the expressed process of IFN-*β*-induced *SOCS3* in astrocytes depended on the activation of *STAT3*. Destruction of the expression of *SOCS3* caused a great deal of inflammatory responses and promoted the migration of microglial and T cells [91]. Shi et al. [92] revealed that the signaling pathway of ER-JAK2/STAT3/SOCS3 was activated by bisphenol F, which propelled the polarization of macrophages to the proinflammatory M1 subtype. Additionally, McFarland et al. [93] found that myeloid cell population without *SOCS3* delayed the growth of intracranial tumors and raised survival rates in the orthotopic glioma-bearing mice. Also, Sheu et al. have found a novel transcriptional mechanism of ECM accumulation induced by high glucose. Specifically, high glucose enhanced *SOCS3* expression via activating PI3K and STAT1/3, which produced a multitude of cascade reactions to form the ECM [94]. Therefore, we believe that *SOCS3* will be a promising prognostic biomarker of patients with glioma.

*SPP1*, a secreted phosphoprotein 1, also called osteopontin (*OPN*), encodes the chemokine-like, calcified ECM-associated proteins. It plays a pivotal role in regulating immune functions and participating in adhesion, remodeling ECM, proliferation, and angiogenesis, as well as metastasis of tumors [95, 96]. Saitoh et al. [97] demonstrated that *OPN* had a close relation to glioma's malignancy. *OPN* is one of the most significant components of the ECM, which is involved in the regulation of matrix interactions and cell adhesion. Also, *OPN* was able to interact with ECM elements, such as collagen, fibronectin, and calcium ion [98]. Besides, Friedmann-Morvinski et al. [99] indicated that silencing of *OPN* significantly exerted influence on the cell cycle and WNT as well as focal adhesion signaling pathways in GBM patients. At the same time, they have drawn a conclusion that *OPN* serves a crucial role in cell dedifferentiation during the formation of tumors. As a consequence, inhibition of *OPN* might be the potential target for the treatment of GBM. Also, *OPN* was overexpressed in almost 90% GBM patients, and a high level of *OPN* was associated with tumor malignancy, where *OPN* recruited neutrophils and macrophages, inducing tumor cell and leukocyte migration [100]. Similarly, macrophages were recruited by *OPN* to the site of GBM, implying that *OPN* acted as a crosstalk between glioma cells and the innate immune system. Thus, *OPN* could be considered to be an outstanding therapeutic target [101].

*TGFB2*, transforming growth factor beta 2, encodes the ligands of the TGF-beta super-family's proteins. These ligands bind a variety of TGF-beta receptors, leading to recruiting *SMAD* transcription factors, which modulate gene expressions. Chen et al. [102] found that malignant glioma cells were able to regulate some adhesion molecules (such as VCAM-1) via secreting TGF-beta2 and releasing the tumor necrosis factor (TNF) receptor. Of note, Corbet et al. [103] demonstrated that *TGF-beta2* was the main driving force of the reconstruction of lipid metabolism promoted by tumor acidosis and was essential to support energy requirements of cancer cells' invasion. Moreover, they also found that acidosis-induced *TGF-beta2* activation promoted partial epithelial-to-mesenchymal transition (EMT) and fatty acid metabolism in various original tumor cells, where the latter supported the acetylation of Smad2. Similarly, *TGF-beta2* activated the autophagy of human glioma cell lines via the Smad and non-Smad pathways, facilitating glioma cell invasion, where epithelial-interstitial transformation and metabolic alterations were vital for glioma progression [104]. Additionally, Xiao et al. [105] showed that *TGF-beta2* functioned as a key factor influencing immune cell recruitment and infiltration to the gastric tumors' site. Therefore, *TGF-beta2* may be regarded as a valuable prognostic biomarker for tumors.

There were some advantages in the present study. First, we adopted the top-ranking 5000 gene expression values based on the MAD algorithm for performing WGCNA, avoiding utilizing DEGs, which was not recommended by the official website. Second, we also removed the batch-batch difference and performed differential expression analysis using the integrated transcriptome analysis based on four GEO datasets, which enabled our results to be more reliable. However, the limitations also need to be given more importance. Given that glioma tissues were difficult to collect, it is challenging to perform experimental validation in gene expressions. Furthermore, it is also rather restricted to construct a model of glioma progression using glioma cell lines for our laboratory. Finally, the specific mechanism and interrelationship of core IRGs in the IRRS model deserve to be further investigated.

## 5. Conclusions

Taken together, in the present study, we established an IRRS prognostic model, composed of *VEGFA*, *SOCS3*, *SPP1*, and *TGFB2* core DE IRGs, for risk stratification and survival prediction for glioma patients. Additionally, glioma patients with high risk stratified by this IRRS prognostic model presented a short survival time, which might be attributed to the ECM signal pathway where those genes participate in. However, a multitude of prospective studies and experiments will be needed to verify our findings in the near future.

## Figures and Tables

**Table 1:** Clusters of PPI network using the MCODE algorithm.

Cluster	Nodes	Edges	Score	Gene symbol
A	18	153	18	*F8*, *FIGF*, *TGFB1*, *VEGFB*, ***TIMP1***, *EGF*, ***TGFB2***, *TGFB3*, *IGF1*, *VEGFC*, *HGF*, ***THBS1***, *ALB*, ***FN1***, *AHSG*, ***VEGFA***, *PDGFB*, and *VWF*
B	16	58	7.733	*CYR61*, ***CUL5***, *SOCS3*, ***IL6***, ***TCEB1***, ***IGFBP1***, *IGFBP3*, *IGFBP5*, ***RBX***, *SPP1*, ***CUL2***, ***IGFBP4***, ***VHL***, ***TCEB2***, *SOCS2*, and ***RNF7***
C	15	52	7.429	*PLCG1*, *PDGFRB*, *STAT5B*, *SYK*, *FLT1*, *VAV1*, *PGF*, *KDR*, *LCP2*, *FYN*, *STAT5A*, *GAB1*, *STAT1*, *PTPN11*, and *EGFR*
D	6	15	6	*RAMP1*, *RAMP2*, ***ADM***, *CALCRL*, *RAMP3*, and ***CALCA***

PPI: protein-protein interaction; MCODE: molecular complex detection; bold texts indicate differentially expressed genes in the PPI network.

**Table 2:** The hazard ratio of the five DE IRGs for evaluating the impact on gliomas' survival.

	TCGA cohort				CGGA cohort			
Genes	HR (95% CI)	5-year survival rates (%)			HR (95% CI)	5-year survival rates (%)		
		Low	High	*P* value		Low	High	*P* value
*VEGFA*	4.03 (3.16-5.15)	67.20	20.38	**<0.0001**	2.62 (1.99-3.45)	67.40	33.70	**2.383** **E** **-12**
*SOCS3*	4.26 (3.35-5.43)	65.60	23.85	**<0.0001**	3.70 (2.80-4.89)	71.40	30.20	**<0.0001**
*THBS1*	2.93 (2.31-3.72)	59.80	29.61	**<0.0001**	2.25 (1.71-2.97)	64.70	36.70	**3.727** **E** **-09**
*SPP1*	3.80 (2.98-4.85)	65.50	24.27	**<0.0001**	2.21 (1.68-2.90)	63.50	38.30	**1.421** **E** **-08**
*TGFB2*	4.64 (3.64-5.92)	67.60	21.93	**<0.0001**	2.26 (1.72-2.97)	65.00	37.60	**6.757** **E** **-09**

TCGA: The Cancer Genome Atlas; CGGA: Chinese Glioma Genome Atlas; HR: hazard ratio; *VEGFA*: vascular endothelial growth factor A; *SOCS3*: suppressor of cytokine signaling 3; *THBS1*: thrombospondin 1; *SPP1*: secreted phosphoprotein 1; *TGFB2*: transforming growth factor beta 2; bold texts indicate statistically significant difference.

**Table 3:** Multivariate Cox regression analysis.

Gene symbol	Description	Coefficient	HR	95% CI	*P* value
*VEGFA*	Vascular endothelial growth factor A	0.20365	1.23	1.12-1.34	1.02**E** − 05^∗∗∗^
*SOCS3*	Suppressor of cytokine signaling 3	0.12448	1.13	1.02-1.25	0.0165^∗^
*SPP1*	Secreted phosphoprotein 1	0.26231	1.30	1.20-1.41	1.10**E** − 09^∗∗∗^
*TGFB2*	Transforming growth factor beta 2	0.1293	1.14	1.02-1.27	0.0168^∗^

HR: hazard ratio; ∗∗∗ and ∗ shows *P* value less than 0.01 and 0.05, respectively; bold texts indicate statistically significant difference.

**Table 4:** The correlations between the risk score and clinicopathological characteristics.

Characteristics	TCGA cohort (*n* = 688)			CGGA cohort (*n* = 422)		
	High risk	Low risk	*P* value	High risk	Low risk	*P* value
Age			**P** < 0.0001			**P** < 0.0001
<60	189	342		159	203	
≥60	120	37		46	13	
NA	—	—		1	—	
Gender			0.8164			0.4308
Female	131	165		83	96	
Male	178	214		123	120	
Tumor grade			**P** < 0.0001			**P** < 0.0001
Low	149	374		93	189	
High	160	5		113	27	
*IDH* status			**P** < 0.0001			**P** < 0.0001
Mutation	78	356		66	142	
Wild-type	223	21		137	38	
NA	8	2		3	36	
*MGMT* promoter status			**P** < 0.0001			0.2748
Methylation	148	339		90	107	
Unmethylation	124	39		76	70	
NA	37	1		40	39	

TCGA: The Cancer Genome Atlas; CGGA: Chinese Glioma Genome Atlas; *IDH*: isocitrate dehydrogenase; *MGMT*: O-6-methylguanine-DNA methyltransferase; NA: not available; bold texts indicate statistically significant difference.

**Table 5:** Univariate and multivariate Cox regression analyses for the IRRS model and clinical traits.

Variables	Univariate cox analysis						Multivariate cox analysis					
	TCGA			CGGA			TCGA			CGGA		
	HR (95% CI)	*P* value	C-index (95% CI)	HR (95% CI)	*P* value	C-index (95% CI)	HR (95% CI)	*P* value	C-index (95% CI)	HR (95% CI)	*P* value	C-index (95% CI)
Age	4.89 (3.772-6.33)	**<0.0001**	0.671 (0.640-0.702)	3.15 (2.269-4.39)	**8.25** **E** **-12**	0.581 (0.554-0.608)	2.02 (1.450-2.81)	**3.20** **E** **-05**	0.847 (0.823-0.871)	1.21 (0.821-1.79)	0.3349	0.765 (0.732-0.798)
Gender	1.26 (0.986-1.61)	0.0645	0.533 (0.500-0.566)	1.16 (0.875-1.53)	0.309	0.506 (0.471-0.541)	—	**—**		—	—	
Grade	9.23 (7.095-12.01)	**<0.0001**	0.736 (0.709-0.763)	4.20 (2.851-6.17)	**3.43** **E** **-13**	0.638 (0.609-0.667)	2.54 (1.750-3.69)	**9.50** **E** **-07**		3.05 (1.916-4.86)	**2.61** **E** **-06**	
Risk score	6.53 (4.960-8.59)	**<0.0001**	0.743 (0.719-0.767)	3.85 (2.857-5.19)	**<0.0001**	0.661 (0.630-0.692)	1.89 (1.252-2.85)	**2.40** **E** **-03**		2.17 (1.493-3.14)	**4.63** **E** **-05**	
*IDH*_status	8.76 (6.708-11.43)	**<0.0001**	0.782 (0.758-0.806)	4.42 (3.278-5.96)	**<0.0001**	0.699 (0.672-0.726)	2.70 (1.700-4.28)	**2.51** **E** **-05**		2.85 (1.966-4.14)	**3.46** **E** **-08**	
*MGMT*_status	3.17 (2.430-4.13)	**<0.0001**	0.654 (0.619-0.689)	1.36 (1.009-1.82)	**0.0434**	0.528 (0.489-0.567)	1.29 (0.938-2.78)	0.1168		1.20 (0.884-1.64)	0.2389	

HR: hazard ratio; IRRS: immune-related risk score; TCGA: The Cancer Genome Atlas; CGGA: Chinese Glioma Genome Atlas; *IDH*: isocitrate dehydrogenase; *MGMT*: O-6-methylguanine-DNA methyltransferase; bold texts indicate statistically significant difference.

**Table 6:** Comparison with other prognostic models of glioma.

First author	Year	Subtype	Prognostic models	AUC (95% CI)			C-index (95% CI)	HR (95% CI)
Bao [60]	2014	Glioma	*RAB1A*, *BIRC5*, *TEAD2*, *TUBA1B*, *MT1E*, *SFXN4*, *TPX2*, *HDAC4*, and *FAM125B*	NA			NA	NA
Bingxiang [61]	2021	GBM	*G6PC2*, *STC1*, *HDAC4*, *COG2*, *SRD5A3*, *MDH2*, *IL13RA1*, *TGFBI*, and *B3GAT3*	1 year: 0.745	3 years: 0.763	—	NA	1.783 (1.496-2.125)
Chen [62]	2016	LGG	*HOXA7*, *SLC2A4RG*, and *MN1*	CGGA: 0.869 (0.774-0.964)	TCGA: 0.785 (0.707-0.863)	—	NA	CGGA: 2.606 (1.690-4.018); TCGA: 3.384 (2.085-5.492)
Fan [63]	2021	GBM	*CLEC5A*, *HOXC6*, *HOXA5*, *CCL2*, *GPRASP1*, *BSCL2*, and *PTX3*	TCGA training: 0.721	TCGA test: 0.688	CGGA: 0.692	NA	TCGA set: 3.77 (2.05-6.92)
Hou [64]	2019	GBM	*CCL7*, *CPNE9*, *GUCA1A*, *HOXA11*, *HOXC11*, *HOXD11*, *INSL3*, *KHDRBS2*, *KRT19*, *MEPE*, *MLPH*, *NELL1*, *TBX5*, and *TMEM233*	TCGA training set: 0.975	CGGA testing set1: 0.907	GSE13041 testing set 2: 0.905	NA	NA
Jia [65]	2021	IDH-mutant glioma	*MTIF3*, *ITGB7*, *IGSF5*, *HNRNPA3P1*, *HMGA1*, *GJA5*, *FANCL*, *FANCB*, *F5*, *ESR2*, *EFNA1*, *DCBLD1*, *CYP3A5*, *CD97*, *CCRL1*, *CCNY*, *CCDC157*, *C4orf3*, *C1orf51*, *C1orf187*, *C18orf10*, *C15orf42*, *AURKAPS1*, *ARF1*, *ANKRD20A3*, *ALS2CR4*, *ALMS1P*, *ADD3*, *ADAMTS7*, *ADAMTS13*, and *ACAD11*	0.9315			NA	NA
Li [66]	2021	LGG	*RFWD3*, *MPHOSPH9*, *WRN*, and *NUP155*	Training set: 1 year: 0.796; 3 years: 0.710; 5 years: 0.601	Validation set: 1 year: 0.668; 3 years: 0.655; 5 years: 0.655	—	NA	NA
Li [67]	2020	Grade II/III glioma	*HCK*, *HAVCR2*, *CD37*, *LPAR5*, *NAGA*, *C1QC*, *FCER1G*, and *AIF1*	TCGA: 0.80			NA	TCGA: 2.713 (1.675-4.394); CGGA part C dataset: 1.958 (1.158-3.310)
Lin [68]	2020	Glioma	*TAGLN2*, *PDPN*, *TIMP1*, and *EMP3*	TCGA: 0.80 (0.76–0.83)	CGGA: 0.72 (0.68–0.76)	—	NA	TCGA: 1.07 (1.06–1.08); CGGA: 1.19 (1.16–2.23)∗
Pan [69]	2020	GBM	*GRIA2* and *RYR3*	GSE16011 dataset: 2 years: 0.671 (0.58-0.762); 3 years: 0.736 (0.653-0.818); 5 years: 0.776 (0.732-0.819)	CGGA: 2 years: 0.675 (0.552-0.799); 3 years: 0.804 (0.751-0.857); 5 years: 0.795 (0.741-0.848)	TCGA: 2 years: 0.634 (0.517-0.75); 3 years: 0.632 (0.458-0.807); 5 years: 0.766 (0.719-0.814)	NA	GEO set: 1.49 (1.01-2.19); CGGA set: 1.83 (1.10-3.05); TCGA set: 1.66 (1.02-2.71)
Qin [70]	2020	GBM	*S100A8*, *CXCL1*, and *IGLL5*	TCGA: 6 months: 0.604; 1 year: 0.657; 2 years: 0.667; 3 years: 0.667; 5 years: 0.667;	CGGA: 6 months: 0.529; 1 year: 0.572; 2 years: 0.592; 3 years: 0.592; 5 years: 0.592;	—	0.791	2.331 (1.486-3.655)
Qu [71]	2020	Glioma	*ULK1*, *ATG10*, *ATG16L2*, *RB1CC1*, *RUBCNL*, *PRKN*, *GSK3B*, *TBC1D5*, *PIK3CB*, and *RAB33B*	1 year: 0.790	3 years: 0.861	5 years: 0.853	NA	1.19 (1.06-1.34)
Tan [72]	2020	Glioma/LGG/GBM	*LPAR5*, *CD163*, *FPR3*, *P2RY12*, *PLAUR*, and *SIGLEC1*	Glioma: TCGA: 0.784; CGGA: 0.736	LGG: TCGA: 0.666; CGGA: 0.683	GBM: TCGA: 0.546; CGGA: 0.622	NA	NA
Tian [73]	2021	Glioma	IGF2BP3, GNS, RANBP17, SMC4, PTTG1, ST6GALNAC1, TET1, and KLB	TCGA: 3 years: 0.91; 5 years: 0.88; 10 years: 0.83	CGGA: 3 years: 0.81; 5 years: 0.83; 10 years: 0.85	—	NA	TCGA: 1.897 (1.147–3.138)
Wang [74]	2021	LGG	*GRID2*, *FOXO1*, *MYC*, *PTK6*, *IKBKE*, *BIRC5*, and *TP73*	Training set: 1 year: 0.901 (0.846-0.957); 3 years: 0.848 (0.796-0.900); 5 years: 0.750 (0.684-0.817)	Validation set 1: 1 year: 0.808 (0.693-0.923); 3 years: 0.802 (0.739-0.865); 5 years: 0.674 (0.594-0.753)	Validation set 2: 1 year: 0.830 (0.750-0.910); 3 years: 0.828 (0.752-0.904); 5 years: 0.755 (0.674-0.836)	NA	Training set: 1.714 (1.325–2.217); validation set 1: 1.287 (1.113–1.489); validation set 2: 1.225 (1.012–1.484)
Wang [22]	2021	Glioma	*FCER1G*, *NOX4*, *TRIM5*, *SOCS1*, *APOBEC3C*, *BIRC5*, *VIM*, *TNC*, *BMP2*, *CMTM3*, *IL24*, *JAG1*, *CALCRL*, *HNF4G*, and *CDK4*	TCGA: 0.886	GSE43378 validation: 0.688	—	TCGA: 0.741	TCGA: 2.461 (1.945-3.113); GSE43378 validation: 6.940 (2.024-23.802)
Yin [23]	2020	LGG	*BIRC5*, *CRLF1*, *GDF15*, *LTF*, *PRLHR*, and *TNFRSF11B*	TCGA: 1 year: 0.89; 3 years: 0.87; 5 years: 0.76	CGGA: 1 year: 0.72; 3 years: 0.78; 5 years: 0.76	—	NA	TCGA: 1.92 (1.50–2.47)
Zhao [75]	2021	GBM	*CD1D*, *PTX3*, *RAC2*, *ESM1*, *MDK*, *TNFSF14*, *IL2RB*, and *OSMR*	0.778			NA	TCGA: 1.269 (1.126–1.430)
Zuo [76]	2019	GBM	*CD79B*, *MAP2K3*, *IMPDH1*, *SLC16A3*, *MPZL3*, and *APOBR*	CGGA: 1 year: 0.699; 2 years: 0.779	TCGA: 1 year: 0.718; 2 years: 0.704	—	NA	CGGA: 2.40 (1.42–4.06); TCGA: 1.70 (1.10–2.63)
The present study	2021	Glioma	*VEGFA*, *SOCS3*, *SPP1*, and *TGFB2*	TCGA: 1 year: 0.86; 3 years: 0.855; 5 years: 0.812	CGGA: 1 year: 0.715; 3 years: 0.772; 5 years: 0.759	—	0.811 (0.786-0.836)	TCGA: 1.89 (1.252-2.85); CGGA: 2.17 (1.493-3.14)

LGG: low-grade glioma; GBM: glioblastoma; IDH: isocitrate dehydrogenase; TCGA: The Cancer Genome Atlas; CGGA: Chinese Glioma Genome Atlas; CI: confidence interval; NA: not available; ^∗^univariable analysis.

## Data Availability

The data that support the findings of this study are available from The Cancer Genome Atlas database (https://portal.gdc.cancer.gov/), GEO database (GSE45921, GSE15824, GSE43378, GSE4290, and GPL570, http://www.ncbi.nlm.nih.gov/geo/), Chinese Glioma Genome Atlas (CGGA) database (http://www.cgga.org.cn/), Immunology Database and Analysis Portal (ImmPort) database (https://www.immport.org/shared/home), and cBioPortal for Cancer Genomics database (http://www.cbioportal.org/).

## Supplementary Materials

Supplementary 1 Supplementary Table 1: the genes in the turquoise module.

Supplementary 2 Supplementary Table 2: the DEGs between HGG and LGG samples.
