# Supplementary material for: Establishment of an Immune-Related Gene Signature for Risk Stratification for Patients with Glioma
Source: Comput Math Methods Med. 2021 Aug 27;2021:2191709. doi: 10.1155/2021/2191709 (PMC8420975; doi:10.1155/2021/2191709)
Supplement: Supplementary 1 — Supplementary Table 1: the genes in the turquoise module. [file 2191709.f1.docx]

| Supplementary Table 1 The genes in turquoise module |
| --- |
| Genes |
| CHI3L1 |
| COL3A1 |
| IGFBP2 |
| COL1A1 |
| COL1A2 |
| ANXA1 |
| TIMP1 |
| CRNDE |
| COL4A1 |
| COL4A2 |
| PDPN |
| GPR17 |
| MIR21///VMP1 |
| SOCS3 |
| SFRP2 |
| MGP |
| RBP1 |
| EMP3 |
| CD163 |
| ADM |
| S100A8 |
| BCAT1 |
| FABP5 |
| LAMB1 |
| KLRC1///KLRC2 |
| DPYD |
| C8orf4 |
| LOC101928916///NNMT |
| VEGFA |
| LYZ |
| COL5A2 |
| CCL2 |
| GBP2 |
| GBP1 |
| PTX3 |
| GNAL |
| TFPI |
| COL6A2 |
| C1R |
| CP |
| CCDC109B |
| CFI |
| LPPR1 |
| LINC00152///LOC101930489///MIR4435-1HG |
| MS4A4A |
| CTHRC1 |
| FAM155A |
| NAMPT |
| TSTD1 |
| ANXA2 |
| KLRC3 |
| LGALS3 |
| NKAIN4 |
| GALNT13 |
| CAV1 |
| LOC100129518///SOD2 |
| TGFB2 |
| SERPINA3 |
| GPNMB |
| HLA-DQB1///LOC101060835 |
| IGFBP3 |
| PDLIM4 |
| C1S |
| SCIN |
| TUBB6 |
| FAM129A |
| PLAT |
| WEE1 |
| SLC2A10 |
| MIR612///NEAT1 |
| FCGBP |
| LUM |
| CTSC |
| FCGR3A///FCGR3B |
| CD93 |
| TNR |
| SMOC1 |
| SUSD5 |
| GBP3 |
| ACSS3 |
| SOX8 |
| SERPINE1 |
| CHI3L2 |
| NARR///RAB34 |
| ANGPT2 |
| CLIC1 |
| IGFBP5 |
| SLC1A1 |
| LMF1 |
| THBS1 |
| RDH10 |
| DLL1 |
| ARPP21 |
| LY96 |
| LOXL1 |
| PLSCR1 |
| TAGLN2 |
| VMP1 |
| MGAT4C |
| MOXD1 |
| FN1 |
| HLA-DQA1///HLA-DQA2///LOC100509457 |
| PDLIM1 |
| MAFF |
| RHOJ |
| CAV2 |
| PLA2G5 |
| CD44 |
| EYA1 |
| VIM |
| TAGLN |
| EMP1 |
| TGFBI |
| EFEMP1 |
| POSTN |
| S100A11 |
| PHLDA2 |
| CXCR4 |
| MSR1 |
| FERMT1 |
| CTNNA3 |
| PROS1 |
| NRP1 |
| SWAP70 |
| DIRAS3 |
| ALOX5AP |
| FABP7 |
| SERPING1 |
| HSPA6 |
| CADM2 |
| CALD1 |
| PPIC |
| AEBP1 |
| PKIB |
| GLIS3 |
| IL13RA2 |
| NPNT |
| CD14 |
| SERPINH1 |
| DUSP6 |
| OSMR |
| CPVL |
| CYR61 |
| FSTL1 |
| SLITRK5 |
| CD58 |
| ATF3 |
| FZD6 |
| LTF |
| RGS1 |
| SCG3 |
| PRSS23 |
| F11R |
| S100A10 |
| SPHKAP |
| RASL10A |
| ZNF217 |
| KIAA0226L |
| C1RL |
| SERPINA1 |
| MYT1 |
| ECM2 |
| LATS2 |
| METTL7B |
| RNASE4 |
| GRIA2 |
| CA12 |
| FLNA |
| IFI44L |
| FAM46A |
| TGFB1I1 |
| LGR5 |
| SEZ6L |
| MYOF |
| OCIAD2 |
| GEM |
| PPM1L |
| C9orf64 |
| OLR1 |
| LAMC1 |
| TGIF1 |
| ACTN1 |
| LOC101927458///LPHN2 |
| SP100 |
| GABBR1 |
| HS3ST3B1 |
| FAM20C |
| ADAM12 |
| LGALS1 |
| PYGL |
| SLC1A6 |
| BCAN |
| B3GNT5 |
| RNF135 |
| TNFSF13B |
| SMIM3 |
| LOXL2 |
| FAS |
| BGN |
| ANXA2P2 |
| HLA-DRA |
| ZAK |
| C3 |
| TNFAIP6 |
| SNAI2 |
| MSN |
| DSCAML1 |
| BICD1 |
| FCER1G |
| SAMD9L |
| ADAM22 |
| MIR6787///SLC16A3 |
| LAMA4 |
| MN1 |
| YBX3 |
| PLP2 |
| CXCL8 |
| CPNE5 |
| FNDC3B///LOC101928615 |
| KCNB1 |
| CD109 |
| CTSS |
| CSMD3 |
| PTGFRN |
| NFKBIZ |
| SLC39A8 |
| EVA1C |
| ITGA2 |
| SOD2 |
| HLA-DQA1///LOC100509457 |
| SEC24D |
| MAOB |
| XAF1 |
| CFH///CFHR1 |
| CARD16///CASP1 |
| CNGA3 |
| GADD45A |
| IKBIP |
| WWTR1 |
| MS4A6A |
| CTNNA1 |
| NET1 |
| CEBPD |
| ST8SIA4 |
| S100A4 |
| GSAP |
| C1QB |
| NAPSB |
| PTRF |
| ELFN2 |
| C1QA |
| PSRC1 |
| PTPRC |
| BTN3A2 |
| WLS |
| SLC25A43 |
| NR2E1 |
| CAPG |
| ARL4C |
| HLA-DQB1///HLA-DRB1///HLA-DRB3///HLA-DRB4///LOC100996809///LOC101060835 |
| RBMS1 |
| RND3 |
| SLC26A2 |
| PDGFA |
| HLA-DQB1///HLA-DRB1///HLA-DRB4///HLA-DRB5///LOC100996809///LOC101060835 |
| PLA2G4A |
| CLIC4 |
| TNFRSF1A |
| ATCAY |
| SPRY4 |
| SFRP4 |
| OSBPL3 |
| C5AR1 |
| ARHGAP18 |
| SCN3A |
| CRTAC1 |
| CPM |
| SAMD9 |
| CASP1 |
| SLC1A4 |
| TMEM176B |
| EFEMP2 |
| HMOX1 |
| VSIG4 |
| LRP1B |
| CA2 |
| HLA-DPA1 |
| TM4SF1 |
| F3 |
| MS4A7 |
| ZNF804A |
| TRIP6 |
| MAN1C1 |
| C10orf10 |
| CD74 |
| SOCS2 |
| LOH12CR1 |
| ITGB1 |
| STEAP3 |
| ITGA7 |
| RNF150 |
| TIFA |
| ABCG1 |
| SPOCD1 |
| SPRY4-IT1 |
| KDELC2 |
| FAM126A |
| AK4///LOC100507855 |
| KDELR2 |
| COL6A3 |
| PLOD2 |
| TUB |
| CTGF |
| SPRY1 |
| S100A6 |
| S1PR3 |
| ZDHHC22 |
| ELTD1 |
| ADORA3 |
| ISG15 |
| FOSL2 |
| GNS |
| YAP1 |
| FKBP9 |
| FLJ16779 |
| RUNX1T1 |
| NPL |
| ADAM9 |
| FYB |
| ALOX5 |
| PLAU |
| NMI |
| MYO1B |
| CNTN1 |
| LINC00925 |
| RHPN2 |
| NEK6 |
| RFTN1 |
| SERPINB1 |
| TNFRSF10B |
| PDGFD |
| CD302///LY75///LY75-CD302 |
| MYL9 |
| TPM2 |
| MDK |
| VAMP8 |
| MCL1 |
| ITGB2 |
| HLA-DRB1///HLA-DRB4///LOC100996809 |
| DUSP5 |
| ENHO |
| ALCAM |
| TOM1L1 |
| HK2 |
| LINC00672 |
| TFRC |
| SPRY2 |
| TMED5 |
| IQGAP1 |
| AHR |
| TWSG1 |
| TOX |
| LDHA |
| CAST |
| NTN4 |
| EIF4E2 |
| CX3CR1 |
| ABCC8 |
| DOCK8 |
| CACNA1A///LOC100507353 |
| HSPB1 |
| SAMSN1 |
| PLEKHG1 |
| ACTA2 |
| SLC25A24 |
| STAB1 |
| FAM19A5 |
| SOAT1 |
| AMER2 |
| DDX25 |
| NID2 |
| EPHB1 |
| GIMAP2 |
| NEDD9 |
| DPY19L1 |
| CALU |
| GBE1 |
| AKAP2///PALM2-AKAP2 |
| TRIM22 |
| GLIPR1 |
| PPP1R9A |
| HLA-B |
| PID1 |
| ELK3 |
| LOC400043 |
| ABRACL |
| SH3GLB1 |
| TRIM5 |
| SRGN |
| PTPN12 |
| BAX |
| CARD16 |
| MX1 |
| CTTNBP2 |
| PIPOX |
| HLA-DQB1 |
| FCGR1B |
| CASP6 |
| SLC16A9 |
| FCGR2A |
| COLEC12 |
| HS2ST1 |
| PSMB9 |
| SEMA6B |
| HIST2H2AA3///HIST2H2AA4 |
| CXXC4 |
| IFI30///PIK3R2 |
| HLA-DRB1///HLA-DRB1 |
| SPP1 |
| JUN |
| DNAJB1 |
| SHC1 |
| GDPD1 |
| SLC43A3 |
| LINC01102 |
| HLA-DPB1 |
| MAFB |
| FAM222A |
| SPRED1 |
| ANTXR2 |
| SLIT1 |
| TMEM64 |
| LRRTM4 |
| LAP3 |
| FAM133A |
| BST2 |
| HLA-DMA |
| MIR7110///PDIA5 |
| TMEM257 |
| PARP9 |
| PABPC1L |
| ARHGDIB |
| SLC16A4 |
| PDK1 |
| SLC40A1 |
| PLS3 |
| IFI6 |
| C21orf62 |
| MCAM///MIR6756 |
| FZD2 |
| TYROBP |
| VAV3 |
| GPX8 |
| DSE |
| FRY |
| SQRDL |
| LOC100506403///LOC101928269///RUNX1 |
| IFI44 |
| TNFAIP8 |
| C1orf85 |
| LINC00537 |
| KIF21B |
| SETD9 |
| CLEC5A |
| TNFRSF12A |
| PPP1R3B |
| HILPDA |
| HLA-C |
| STAT1 |
| C1orf54 |
| RICTOR |
| GNG4 |
| NOG |
| KLF10 |
| JAM3 |
| RUNX2 |
| PAICS |
| HOTAIRM1 |
| HES1 |
| TP73-AS1 |
| UPP1 |
| CNIH4 |
| P4HB |
| CLEC7A |
| LRRFIP1 |
| AMOTL2 |
| IL13RA1 |
| GALNT2 |
| ETNK1 |
| BLNK |
| EYA2 |
| LXN |
| ENPEP |
| GCLM |
| SYNC |
| MICALL2 |
| MNDA |
| CSTA |
| OLFML2B |
| PLXDC1 |
| LIMK2 |
| KANK2 |
| TRAF3IP2-AS1 |
| HERC5 |
| PCOLCE2 |
| DCTD |
| IFI16 |
| CRYZ |
| PGM2 |
| LAMB2 |
| GALNT4///POC1B-GALNT4 |
| PDLIM3 |
| RECQL |
| RHOQ |
| LPAR6 |
| GNG5 |
| ALDH5A1 |
| RCAN1 |
| GRIK2 |
| MAPT |
| FGL2 |
| FAM26F |
| WIPI1 |
| ARHGAP29 |
| EFNB2 |
| IER3 |
| TRAF5 |
| SLC8A3 |
| C1QC |
| SEC61A1 |
| VAMP5 |
| MCAM |
| LACTB2 |
| GPR34 |
| WDR1 |
| PRPS2 |
| PSMB8 |
| LOC100996792///MAP2K3 |
| ANO6 |
| P2RX7 |
| HCLS1 |
| PLIN2 |
| RARRES3 |
| DPH3 |
| LOX |
| RGS10 |
| CTBS |
| CNRIP1 |
| STC1 |
| TEAD2 |
| TP53I3 |
| CDH10 |
| HIPK2 |
| LAMP2 |
| IFITM3 |
| TGFBR2 |
| OLFML3 |
| TRAM1 |
| EHD4 |
| PDGFC |
| SLC27A3 |
| HIP1R |
| RPRM |
| LAPTM5 |
| CSDC2 |
| RAP2A |
| GNG12 |
| CPQ |
| UCP2 |
| NES |
| PINLYP |
| PQLC3 |
| PARP12 |
| FAM111A |
| FNBP1L |
| ANXA5 |
| LCP2 |
| GLUD1 |
| AP1S2 |
| FAM114A1 |
| SPIDR |
| RNFT1 |
| SLC16A1 |
| LMO2 |
| RBM47 |
| TMEM185B |
| RNASE6 |
| DTX3L |
| MXRA5 |
| LOC100506548///RPL37 |
| MBD2 |
| RELL1 |
| BTG3 |
| TMEM119 |
| KLHL4 |
| C2orf27A |
| GLUD2 |
| LBH |
| UGCG |
| IBSP |
| CASP8 |
| EMILIN1 |
| LYN |
| RARRES2 |
| FCGR1A///FCGR1B///FCGR1C |
| SPPL2A |
| TPM4 |
| ELOVL6 |
| HEXB |
| KCNK3 |
| RREB1 |
| DENND2A |
| ASXL3 |
| SLC35F5 |
| CBR1 |
| H2BFS |
| PGK1 |
| EVI2B |
| NRXN2 |
| C1orf53 |
| REXO2 |
| COL12A1 |
| VWF |
| RASSF8 |
| PLEKHA4 |
| FERMT2 |
| HLA-F |
| GRB10 |
| ELN |
| ERI1 |
| GLYR1///SEPT6 |
| MAPK10 |
| CYBA |
| MIR22///MIR22HG |
| LMNA |
| NSUN6 |
| CRTAP |
| IL6ST |
| CD37 |
| FKBP10 |
| OAS1 |
| ARPC1B |
| IGFBP7 |
| PDZD8 |
| RAB27A |
| SLC2A5 |
| TRIM14 |
| FPR1 |
| SSFA2 |
| CLU |
| LGALS3BP |
| HRH1 |
| COTL1 |
| FBLN5 |
| THOC2 |
| LRRC4C |
| LEF1 |
| FUCA2 |
| DDB2 |
| SLC38A6 |
| PCSK5 |
| TSPO |
| HDAC4 |
| BHLHE40 |
| LRRTM2 |
| GTF2I |
| LPP |
| WISP1 |
| GUSB |
| KCNIP2 |
| ST6GAL2 |
| CD164 |
| KLF6 |
| LUZP2 |
| CHST6 |
| RBPMS |
| PLCE1 |
| SATB1 |
| LPAR5 |
| FLNC |
| PRDX4 |
| ITPRIPL2 |
| SLC11A1 |
| TRIM56 |
| IGFBP4 |
| C12orf5 |
| IER5 |
| MGC12488 |
| SP140L |
| MRC2 |
| APBB1IP |
| IRF7 |
| BHLHE41 |
| BTN3A3 |
| CYBRD1 |
| CNN3 |
| APOC1 |
| LPCAT2 |
| SS18 |
| ABCD3 |
| PDIA4 |
| IMPACT |
| RASSF3 |
| MAPT-AS1 |
| SLC25A48 |
| RNASET2 |
| COL6A1 |
| COL8A1 |
| REST |
| TM9SF3 |
| SHROOM3 |
| PECAM1 |
| MR1 |
| GNG11 |
| NFIL3 |
| UQCRB |
| IFRD1 |
| TMOD3 |
| LSAMP |
| CEBPG |
| IDI1 |
| GLS |
| METRNL |
| VCL |
| 8-Mar |
| C1orf162 |
| PLTP |
| TMSB10 |
| TMX1 |
| POU6F1 |
| USH1C |
| MIR6513///TMBIM1 |
| SDF4 |
| SAT1 |
| PLXDC2 |
| TCEA3 |
| KDELR1 |
| P2RX5-TAX1BP3///TAX1BP3 |
| CHSY1 |
| CD86 |
| SVIL |
| FAM110B |
| GDF15 |
| FZD1 |
| RABEP2 |
| GNAI3 |
| SRPX2 |
| FILIP1L |
| TP53 |
| TPST1 |
| SLC44A3 |
| LOC102725271///NTM |
| F13A1 |
| DDIT4L |
| DAB2///LOC101926921 |
| CPD |
| SLC39A14 |
| TYMP |
| OSTC |
| XRN2 |
| SSR3 |
| MANEA |
| LARP4 |
| RIT1 |
| RHOC |
| ABCC3 |
| ARSD |
| TMEM248 |
| FDFT1 |
| KLF3 |
| CAMK2D |
| PWAR5 |
| ARRDC3 |
| FPGT |
| CD9 |
| TAP1 |
| TMEM209 |
| KCNJ10 |
| HIPK3 |
| SERP2 |
| EIF4A2///MIR1248///SNORA4///SNORA63///SNORA81///SNORD2 |
| SLC30A7 |
| TGFB1 |
| DNAJC10 |
| DGKI |
| LGALS8 |
| ARHGAP5 |
| LOC101928189///RSRP1 |
| AKAP6 |
| UNC79 |
| IL1R1 |
| CD99 |
| PTGS1 |
| ENAH |
| YTHDF3 |
| MYADM |
| ITGAM |
| KCNIP3 |
| UACA |
| ETV6 |
| ELL2 |
| P2RX5///P2RX5-TAX1BP3///TAX1BP3 |
| MYD88 |
| LPHN3 |
| CHST2 |
| TGFBR1 |
| COL18A1 |
| LYPLA1 |
| GLRX |
| ARF4 |
| TREM2 |
| GDAP1L1 |
| DRAM1 |
| CEBPB |
| IFI35 |
| LAMA2 |
| HAMP |
| ADAM28 |
| SPRED2 |
| DOCK7 |
| MMP9 |
| MGME1 |
| CD97 |
| TLR1 |
| GFRA1 |
| HEBP2 |
| CFLAR |
| HLA-G |
| SMAD7 |
| TXLNA |
| SEPT2 |
| MIR4745///PTBP1 |
| IPW///LOC101930404///PWARSN///SNORD107///SNORD115-13///SNORD115-26///SNORD115-7///SNORD116-22///SNORD116-28///SNORD116-4///SNRPN |
| NPC2 |
| ATOH8 |
| RAB8B |
| CYBB |
| NPEPL1 |
| ENO1 |
| NOTCH3 |
| PDIA6 |
| IFIH1 |
| CCSER2 |
| DPYSL4 |
| IFITM2 |
| MAGOH///MAGOHB |
| CMTM6 |
| PTGR1 |
| RNF165 |
| ARMCX4 |
| DNALI1 |
| CTSB |
| TWF1 |
| NDUFA4L2 |
| SLC2A14///SLC2A3 |
| SNAP23 |
| KDELR3 |
| ZC3H12B |
| CDH5 |
| DDOST |
| GPR123 |
| CD151 |
| PALLD |
| ADCY7 |
| HIST1H2BD |
| LIMS1///LIMS3///LIMS3L |
| MRPS12 |
| WIPF1 |
| APOBEC3G |
| PTGER4 |
| TNK2 |
| KIF13A |
| PRR24 |
| TNFAIP3 |
| RER1 |
| KIAA0040 |
| PPIB |
| MKX |
| RPAP3 |
| ATP9A |
| HAT1 |
| RNF149 |
| FRMD8 |
| PTBP1 |
| ACTL6A |
| SOCS6 |
| MGAT4B |
| PSENEN |
| LRCH2 |
| CCDC50 |
| CMTM3 |
| MOB1A |
| PFN1 |
| GCH1 |
| PAPOLA |
| MEIS3P1 |
| CSF1R |
| SIPA1L2 |
| HSPG2 |
| SORD |
| STARD10 |
| PHLPP1 |
| RRP15 |
| HSPA5 |
| NEDD1 |
| FLRT1 |
| SYK |
| RBM17 |
| GSN |
| BCR |
| EOGT |
| TSPAN4 |
| ASTN1 |
| PRPF31 |
| AGTRAP |
| MREG |
| COL15A1 |
| SGMS2 |
| HCK |
| CHD3 |
| DDX39A |
| KCNE3 |
| ZMYM6///ZMYM6NB |
| NOX4 |
| CACNG2 |
| GIMAP6 |
| EMILIN2 |
| SAP30 |
| CKLF///CKLF-CMTM1 |
| ANXA4 |
| LITAF |
| ICAM1 |
| KHNYN |
| LOC154761 |
| RRM1 |
| FCHSD2 |
| ITGA4 |
| ZCCHC24 |
| NTNG2 |
| TEP1 |
| PTPLAD2 |
| TNFSF10 |
| PITPNC1 |
| S100A16 |
| KISS1R |
| CALR |
| MTMR11 |
| ITGB8 |
| YKT6 |
| LOC100129034 |
| RUFY3 |
| CECR1 |
| RIPPLY2 |
| DRAM2 |
| ARPC5 |
| FADS1///MIR1908 |
| RRBP1 |
| ACOT9 |
| VASN |
| PCOLCE |
| C15orf59 |
| APOC2///APOC4///APOC4-APOC2 |
| ANKRD22 |
| PMP22 |
| VAMP3 |
| ITGA5 |
| CD2AP |
| SMS |
| COL5A1 |
| BCL2A1 |
| SERPINB6 |
| ELAVL1 |
| FBXO17 |
| HSPA4 |
| MAPK8 |
| MVP |
| MDGA2 |
| NCKAP1L |
| PSMB8-AS1 |
| TIMP2 |
| SLC25A37 |
| DUSP4 |
| ADAM10 |
| TXNDC17 |
| COQ2 |
| PRKD2 |
| PHF11 |
| MYL12A |
| KCTD9 |
| GJA9///GJA9-MYCBP///MYCBP |
| DR1 |
| CD47 |
| MEOX2 |
| GNL2 |
| ISG20 |
| HLA-J |
| IL10RA |
| CASP7 |
| CKLF |
| TNPO1 |
| MERTK |
| CA13///LOC100507258 |
| RPS15A |
| CD63 |
| TM4SF18 |
| RSAD2 |
| DCBLD2 |
| IPW///LOC101930404///PWARSN///SNORD107///SNORD115-13///SNORD115-26///SNORD115-7///SNORD116-22///SNORD116-28///SNORD116-4 |
| AGAP4 |
| FXYD6 |
| NUP37 |
| HIST1H2BK |
| CDR2///LOC101060399 |
| SLC4A7 |
| RPS27L |
| G0S2 |
| SLC2A3 |
| DNAJC2 |
| C14orf142 |
| FMOD |
| HLA-A |
| RMDN1 |
| ACAA2 |
| RTCA |
| MMP14 |
| GYS1 |
| ATP10D |
| KAT6B |
| KCNE4 |
| DHRS9 |
| BZW1 |
| RAB29 |
| CELSR3 |
| ZBTB18 |
| PTPN13 |
| CLCC1 |
| LOC101927705///P4HA2 |
| BTN3A2///BTN3A3 |
| MTSS1 |
| ACLY |
| PARP14 |
| FAF1 |
| FGFRL1 |
| OLFML2A |
| GSTK1 |
| CFH |
| AIF1 |
| CLEC18A///CLEC18C |
| FEM1C |
| NRAS |
| BBIP1 |
| FBXO22 |
| SEPT10 |
| TNFSF13 |
| ABAT |
| PDZD4 |
| PCYT1B |
| PTPN14 |
| TRIM6 |
| ARAP3 |
| GAREML |
| LOC283075 |
| DDX60L |
| TMEM167A |
| GPR65 |
| PHTF1 |
| CKAP4 |
| A2M |
| TCIRG1 |
| ENTPD1 |
| NFATC2 |
| LHFPL2 |
| TM6SF1 |
| NLRC5 |
| ZRANB1 |
| HSP90B1///MIR3652 |
| KBTBD2 |
| MAP2 |
| POLR2L |
| SSR1 |
| CCR1 |
| ESM1 |
| KIAA1161 |
| TPGS2 |
| FCGR3B |
| HAVCR2 |
| LRP10 |
| ENG |
| HLA-E |
| CANX |
| BACE2 |
| PLIN3 |
