# Supplementary material for: Establishment of an Immune-Related Gene Signature for Risk Stratification for Patients with Glioma
Source: Comput Math Methods Med. 2021 Aug 27;2021:2191709. doi: 10.1155/2021/2191709 (PMC8420975; doi:10.1155/2021/2191709)
Supplement: Supplementary 2 — Supplementary Table 2: the DEGs between HGG and LGG samples. [file 2191709.f2.docx]

| Supplementary Table 2 The DEGs between HGG and LGG samples | | | | | | | |
| --- | --- | --- | --- | --- | --- | --- | --- |
| ID | Gene | log2FC | AveExpr | t | P.Value | adj.P.Val | B |
| 209395_at | CHI3L1 | 3.250574003 | 9.930370989 | 9.142887584 | 2.12E-17 | 2.24E-14 | 28.89865909 |
| 202718_at | IGFBP2 | 2.5075951 | 8.953588957 | 10.53097431 | 1.01E-21 | 1.53E-17 | 38.57482428 |
| 1556499_s_at | COL1A1 | 2.367236155 | 9.100479103 | 7.640753663 | 4.54E-13 | 4.65E-11 | 19.22248503 |
| 201012_at | ANXA1 | 2.363646332 | 9.794449569 | 9.563846033 | 1.11E-18 | 2.52E-15 | 31.76920063 |
| 215076_s_at | COL3A1 | 2.363296069 | 8.559132676 | 7.964012866 | 5.77E-14 | 8.45E-12 | 21.22129613 |
| 223122_s_at | SFRP2 | -2.33073385 | 8.204837483 | -8.641665713 | 6.56E-16 | 2.91E-13 | 25.56498287 |
| 221008_s_at | ETNPPL | -2.322438406 | 8.862640911 | -7.496761839 | 1.12E-12 | 9.66E-11 | 18.34851341 |
| 202237_at | LOC101928916///NNMT | 2.321317121 | 8.209012535 | 8.246275114 | 9.15E-15 | 2.07E-12 | 23.00629653 |
| 201666_at | TIMP1 | 2.305573545 | 10.25986575 | 9.838419544 | 1.56E-19 | 6.56E-16 | 33.67322483 |
| 202404_s_at | COL1A2 | 2.170975608 | 8.321830345 | 8.262033252 | 8.25E-15 | 1.91E-12 | 23.10699689 |
| 210809_s_at | POSTN | 2.127689242 | 7.734104135 | 6.065559756 | 4.80E-09 | 1.04E-07 | 10.27535472 |
| 211980_at | COL4A1 | 2.035285841 | 10.03955415 | 9.356272244 | 4.78E-18 | 7.69E-15 | 30.34610759 |
| 202018_s_at | LTF | 2.010684746 | 8.356724609 | 6.644817618 | 1.87E-10 | 6.78E-09 | 13.40099788 |
| 238021_s_at | CRNDE | 1.973456339 | 7.633035307 | 8.411411956 | 3.07E-15 | 9.27E-13 | 24.06692234 |
| 206157_at | PTX3 | 1.899425104 | 6.877355207 | 7.480545755 | 1.24E-12 | 1.05E-10 | 18.25073955 |
| 227697_at | SOCS3 | 1.88738851 | 6.875409786 | 8.378571348 | 3.82E-15 | 1.08E-12 | 23.85506352 |
| 211964_at | COL4A2 | 1.883773291 | 10.22194832 | 9.525756197 | 1.45E-18 | 3.05E-15 | 31.50698318 |
| 221898_at | PDPN | 1.876520713 | 8.24257382 | 8.644415996 | 6.44E-16 | 2.89E-13 | 25.58300809 |
| 202376_at | SERPINA3 | 1.874976337 | 11.30990566 | 8.31227132 | 5.92E-15 | 1.48E-12 | 23.42875932 |
| 206785_s_at | KLRC1///KLRC2 | -1.867401954 | 7.159455901 | -6.326895419 | 1.14E-09 | 3.11E-08 | 11.66013399 |
| 203729_at | EMP3 | 1.864223974 | 8.600535348 | 9.2064647 | 1.36E-17 | 1.66E-14 | 29.32823298 |
| 206201_s_at | MEOX2 | 1.862396664 | 5.610711245 | 6.800684368 | 7.55E-11 | 3.14E-09 | 14.27620473 |
| 227361_at | HS3ST3B1 | 1.854302819 | 6.618677887 | 7.819361726 | 1.46E-13 | 1.83E-11 | 20.32071078 |
| 203820_s_at | IGF2BP3 | 1.802285028 | 6.925882206 | 7.803464408 | 1.62E-13 | 1.99E-11 | 20.22233809 |
| 232010_at | FSTL5 | -1.799165705 | 6.719087646 | -8.638345958 | 6.71E-16 | 2.96E-13 | 25.54322942 |
| 201292_at | TOP2A | 1.799057843 | 7.771858517 | 8.202410898 | 1.22E-14 | 2.56E-12 | 22.726562 |
| 240228_at | CSMD3 | -1.793640502 | 7.466113939 | -10.77187533 | 1.70E-22 | 4.64E-18 | 40.30970481 |
| 230698_at | CALN1 | -1.782841051 | 6.634001082 | -8.137261041 | 1.87E-14 | 3.64E-12 | 22.31265497 |
| 224917_at | MIR21///VMP1 | 1.779213111 | 9.139930104 | 9.056109849 | 3.87E-17 | 3.36E-14 | 28.31469015 |
| 229831_at | CNTN3 | -1.777515367 | 6.909856475 | -7.188842518 | 7.44E-12 | 4.58E-10 | 16.51506035 |
| 210512_s_at | VEGFA | 1.756534339 | 10.4238872 | 9.138448587 | 2.19E-17 | 2.24E-14 | 28.86872035 |
| 210135_s_at | SHOX2 | 1.751212136 | 5.992946497 | 8.683998541 | 4.93E-16 | 2.36E-13 | 25.84276764 |
| 202345_s_at | FABP5 | 1.738274526 | 9.726987253 | 7.593172329 | 6.12E-13 | 5.91E-11 | 18.93254378 |
| 209909_s_at | TGFB2 | 1.731881718 | 6.448942829 | 9.322016582 | 6.08E-18 | 8.83E-15 | 30.11266407 |
| 210095_s_at | IGFBP3 | 1.696695158 | 9.607552864 | 8.358071973 | 4.37E-15 | 1.19E-12 | 23.7230526 |
| 221730_at | COL5A2 | 1.696120142 | 7.500247614 | 9.24451708 | 1.04E-17 | 1.33E-14 | 29.58603407 |
| 202912_at | ADM | 1.684927728 | 8.888610154 | 8.559448692 | 1.14E-15 | 4.54E-13 | 25.0275554 |
| 204563_at | SELL | -1.679782868 | 8.009137452 | -8.117831817 | 2.12E-14 | 4.03E-12 | 22.18958498 |
| 213841_at | TNR | -1.676633685 | 10.57523714 | -8.111013025 | 2.22E-14 | 4.13E-12 | 22.14643308 |
| 228509_at | SPHKAP | -1.65783332 | 8.087140985 | -9.603731904 | 8.33E-19 | 2.07E-15 | 32.0442922 |
| 218802_at | CCDC109B | 1.655921839 | 7.835120265 | 9.851929043 | 1.42E-19 | 6.56E-16 | 33.76751563 |
| 201860_s_at | PLAT | 1.65365151 | 8.40022186 | 9.012259903 | 5.23E-17 | 4.15E-14 | 28.02065662 |
| 231577_s_at | GBP1 | 1.651898708 | 8.483298029 | 8.175003239 | 1.46E-14 | 2.95E-12 | 22.55220689 |
| 225681_at | CTHRC1 | 1.650034113 | 7.726147528 | 6.875494408 | 4.86E-11 | 2.19E-09 | 14.70120017 |
| 218541_s_at | C8orf4 | 1.637169199 | 6.509195227 | 8.301510767 | 6.36E-15 | 1.58E-12 | 23.3597484 |
| 201890_at | RRM2 | 1.635414414 | 7.86197566 | 7.796986011 | 1.68E-13 | 2.06E-11 | 20.18228432 |
| 227690_at | GABRB3 | -1.627626377 | 7.437595107 | -7.687784468 | 3.37E-13 | 3.68E-11 | 19.5101665 |
| 206172_at | IL13RA2 | 1.622270834 | 7.193512632 | 6.104280639 | 3.89E-09 | 8.70E-08 | 10.47782707 |
| 213060_s_at | CHI3L2 | 1.615025456 | 8.022825053 | 7.249016562 | 5.16E-12 | 3.43E-10 | 16.86946552 |
| 60474_at | FERMT1 | -1.613586645 | 8.391352804 | -7.873219322 | 1.03E-13 | 1.39E-11 | 20.654874 |
| 226517_at | BCAT1 | 1.612211753 | 8.668313092 | 8.048785185 | 3.33E-14 | 5.55E-12 | 21.75360228 |
| 238756_at | GAS2L3 | 1.603340888 | 6.947535052 | 9.188106864 | 1.55E-17 | 1.84E-14 | 29.20404491 |
| 204465_s_at | INA | -1.598955845 | 8.903513183 | -7.095324988 | 1.31E-11 | 7.30E-10 | 15.96811221 |
| 228776_at | GJC1 | 1.594749387 | 8.133318269 | 10.88492272 | 7.32E-23 | 4.00E-18 | 41.12864614 |
| 1569003_at | VMP1 | 1.594298782 | 8.371399834 | 8.931418247 | 9.12E-17 | 6.16E-14 | 27.48046103 |
| 219918_s_at | ASPM | 1.591678786 | 6.514509359 | 8.65517611 | 5.99E-16 | 2.80E-13 | 25.65355871 |
| 215446_s_at | LOX | 1.584456365 | 6.048468722 | 6.170526665 | 2.71E-09 | 6.44E-08 | 10.82642692 |
| 204953_at | SNAP91 | -1.577150305 | 8.899927357 | -7.297395746 | 3.84E-12 | 2.67E-10 | 17.15578949 |
| 205751_at | SH3GL2 | -1.570580922 | 8.586197566 | -6.435886292 | 6.17E-10 | 1.87E-08 | 12.25011642 |
| 230869_at | FAM155A | -1.566051226 | 7.017114765 | -8.540358342 | 1.30E-15 | 4.89E-13 | 24.90316337 |
| 225799_at | LINC00152///LOC101930489///MIR4435-1HG | 1.556255299 | 8.31479594 | 9.403972176 | 3.42E-18 | 6.23E-15 | 30.6718431 |
| 201505_at | LAMB1 | 1.555912869 | 6.794099594 | 7.654407565 | 4.16E-13 | 4.32E-11 | 19.3058925 |
| 202291_s_at | MGP | 1.545617972 | 8.325038133 | 6.585462428 | 2.63E-10 | 9.01E-09 | 13.07141901 |
| 1555938_x_at | VIM | 1.545506842 | 6.400518463 | 8.295450436 | 6.62E-15 | 1.63E-12 | 23.32090362 |
| 219148_at | PBK | 1.545031424 | 7.295360718 | 7.436315348 | 1.63E-12 | 1.32E-10 | 17.98473344 |
| 241805_at | GABRG1 | -1.533936223 | 6.463806243 | -7.73033738 | 2.57E-13 | 2.93E-11 | 19.77138741 |
| 229294_at | JPH3 | -1.529096892 | 7.77300122 | -7.758589351 | 2.15E-13 | 2.51E-11 | 19.94530325 |
| 217739_s_at | NAMPT | 1.52846929 | 9.500988321 | 8.695848429 | 4.55E-16 | 2.24E-13 | 25.92065451 |
| 236028_at | IBSP | 1.524472818 | 5.952767153 | 6.164235528 | 2.80E-09 | 6.63E-08 | 10.79320304 |
| 204646_at | DPYD | 1.51757617 | 8.088437001 | 8.0833616 | 2.66E-14 | 4.65E-12 | 21.97165922 |
| 227055_at | METTL7B | 1.500978096 | 9.032294502 | 8.287586718 | 6.97E-15 | 1.68E-12 | 23.27052345 |
| 203645_s_at | CD163 | 1.494943447 | 8.354433334 | 6.682379255 | 1.50E-10 | 5.60E-09 | 13.61062746 |
| 219230_at | TMEM100 | -1.493325193 | 9.306052331 | -6.453941828 | 5.57E-10 | 1.72E-08 | 12.34854772 |
| 203423_at | RBP1 | 1.482944273 | 8.559452327 | 6.81612199 | 6.89E-11 | 2.93E-09 | 14.36364635 |
| 226021_at | RDH10 | 1.480771159 | 7.921851776 | 8.550904543 | 1.21E-15 | 4.67E-13 | 24.97186348 |
| 212097_at | CAV1 | 1.479456554 | 8.995941856 | 8.085775839 | 2.62E-14 | 4.59E-12 | 21.98690492 |
| 222784_at | SMOC1 | -1.472478612 | 10.05507423 | -8.36417699 | 4.20E-15 | 1.17E-12 | 23.76234861 |
| 239468_at | MKX | -1.470680691 | 5.034067518 | -9.770979049 | 2.53E-19 | 8.64E-16 | 33.20335791 |
| 1568612_at | GABRG2 | -1.467688279 | 7.070423873 | -5.196662724 | 4.20E-07 | 4.92E-06 | 5.992819412 |
| 231029_at | F5 | -1.466957055 | 5.565318486 | -6.81505193 | 6.94E-11 | 2.94E-09 | 14.35758096 |
| 217967_s_at | FAM129A | 1.465379269 | 8.165507098 | 8.222755338 | 1.07E-14 | 2.34E-12 | 22.85619894 |
| 210016_at | MYT1L | -1.462995516 | 7.42687075 | -5.11791763 | 6.14E-07 | 6.83E-06 | 5.630507812 |
| 227202_at | CNTN1 | -1.459753451 | 8.330569853 | -6.315323945 | 1.21E-09 | 3.29E-08 | 11.5979212 |
| 235118_at | CADM2 | -1.452380721 | 8.964743496 | -6.801539364 | 7.51E-11 | 3.13E-09 | 14.28104405 |
| 209191_at | TUBB6 | 1.445498567 | 8.349419982 | 8.397943871 | 3.36E-15 | 9.97E-13 | 23.97998259 |
| 236333_at | BC045805 | -1.444165684 | 8.28285947 | -8.13969702 | 1.84E-14 | 3.60E-12 | 22.32809707 |
| 243779_at | GALNT13 | -1.4415457 | 6.616644565 | -9.719063692 | 3.67E-19 | 1.05E-15 | 32.84262104 |
| 212533_at | WEE1 | 1.435430089 | 8.958920756 | 8.737492049 | 3.43E-16 | 1.84E-13 | 26.19481335 |
| 207723_s_at | KLRC3 | -1.431424829 | 6.649449624 | -6.951863267 | 3.09E-11 | 1.50E-09 | 15.13829505 |
| 223557_s_at | TMEFF2 | -1.430132295 | 8.520023665 | -5.989963938 | 7.22E-09 | 1.46E-07 | 9.882817912 |
| 225911_at | NPNT | 1.429283856 | 7.492914425 | 7.282478043 | 4.20E-12 | 2.88E-10 | 17.06737052 |
| 218039_at | NUSAP1 | 1.428265143 | 8.780865657 | 7.807041796 | 1.58E-13 | 1.95E-11 | 20.24446448 |
| 209156_s_at | COL6A2 | 1.427401458 | 7.91984858 | 6.534940905 | 3.51E-10 | 1.15E-08 | 12.79252366 |
| 215223_s_at | LOC100129518///SOD2 | 1.42700611 | 8.390042537 | 8.42004928 | 2.90E-15 | 9.05E-13 | 24.12271883 |
| 222848_at | CENPK | 1.426191218 | 5.76672704 | 8.513189787 | 1.56E-15 | 5.61E-13 | 24.72639352 |
| 214046_at | FUT9 | -1.42610535 | 9.185466124 | -6.47977882 | 4.81E-10 | 1.51E-08 | 12.48974084 |
| 236308_at | VSTM2A | -1.422762389 | 6.845382676 | -5.961806259 | 8.40E-09 | 1.67E-07 | 9.737544941 |
| 219537_x_at | DLL3 | -1.422283577 | 8.638130554 | -6.543453101 | 3.35E-10 | 1.11E-08 | 12.839408 |
| 209160_at | AKR1C3 | -1.41252027 | 8.176373543 | -7.386759948 | 2.21E-12 | 1.67E-10 | 17.68788925 |
| 1554593_s_at | SLC1A6 | -1.411304125 | 5.673369494 | -8.339894559 | 4.93E-15 | 1.30E-12 | 23.60614512 |
| 208636_at | ACTN1 | 1.410329702 | 10.41883019 | 9.990843547 | 5.21E-20 | 3.16E-16 | 34.74028128 |
| 206850_at | RASL10A | -1.408656091 | 8.642872605 | -9.483989709 | 1.95E-18 | 3.80E-15 | 31.22000708 |
| 213308_at | SHANK2 | -1.407269773 | 7.952159844 | -8.102902518 | 2.34E-14 | 4.24E-12 | 22.09513398 |
| 219196_at | SCG3 | -1.403141798 | 9.026652832 | -7.815600589 | 1.50E-13 | 1.86E-11 | 20.29742597 |
| 226777_at | ADAM12 | 1.40236411 | 6.949747627 | 8.787656736 | 2.44E-16 | 1.35E-13 | 26.52598121 |
| 230577_at | LINC00844 | -1.400791645 | 9.528081066 | -6.12119311 | 3.55E-09 | 8.06E-08 | 10.56656087 |
| 228640_at | PCDH7 | -1.400371335 | 8.928550337 | -6.376871558 | 8.60E-10 | 2.47E-08 | 11.92976556 |
| 231935_at | ARPP21 | -1.394742496 | 7.298026078 | -9.851655588 | 1.42E-19 | 6.56E-16 | 33.76560647 |
| 228598_at | DPP10 | -1.392827214 | 7.169498348 | -7.099814723 | 1.27E-11 | 7.14E-10 | 15.99426328 |
| 229799_s_at | NCAM1 | -1.391119079 | 8.45944348 | -8.122450733 | 2.06E-14 | 3.94E-12 | 22.2188271 |
| 218623_at | HMP19 | -1.387383401 | 9.196637366 | -7.69586541 | 3.20E-13 | 3.54E-11 | 19.55970549 |
| 214071_at | GNAL | -1.385471036 | 6.463115104 | -8.171633548 | 1.49E-14 | 3.00E-12 | 22.53079346 |
| 201744_s_at | LUM | 1.378132331 | 6.290166909 | 6.067304463 | 4.75E-09 | 1.03E-07 | 10.28445734 |
| 204260_at | CHGB | -1.375976828 | 7.759894881 | -6.940720012 | 3.30E-11 | 1.59E-09 | 15.07431432 |
| 201324_at | EMP1 | 1.372732127 | 9.525049316 | 7.167564746 | 8.46E-12 | 5.05E-10 | 16.39020234 |
| 230964_at | FREM2 | 1.369064906 | 6.212482841 | 7.092670285 | 1.33E-11 | 7.40E-10 | 15.95265468 |
| 201664_at | SMC4 | 1.36764947 | 8.646553036 | 9.731169367 | 3.36E-19 | 1.05E-15 | 32.92666235 |
| 229823_at | RIMS2 | -1.365934331 | 7.327602005 | -6.104830016 | 3.88E-09 | 8.67E-08 | 10.48070662 |
| 230551_at | KSR2 | -1.365821693 | 7.001713126 | -8.491940333 | 1.79E-15 | 6.24E-13 | 24.5883492 |
| 218883_s_at | CENPU | 1.363901262 | 8.07372496 | 7.759448501 | 2.14E-13 | 2.50E-11 | 19.95059811 |
| 229802_at | WISP1 | 1.363368268 | 5.7935379 | 6.564948523 | 2.96E-10 | 9.98E-09 | 12.95799367 |
| 228033_at | E2F7 | 1.362930585 | 6.098668839 | 8.116524991 | 2.14E-14 | 4.05E-12 | 22.18131326 |
| 223381_at | NUF2 | 1.361581677 | 6.100445341 | 7.808647003 | 1.56E-13 | 1.93E-11 | 20.25439478 |
| 207447_s_at | MGAT4C | -1.354943957 | 5.390635407 | -8.460495089 | 2.21E-15 | 7.24E-13 | 24.38441487 |
| 229725_at | ACSL6 | -1.353802895 | 8.391830481 | -7.354139014 | 2.71E-12 | 2.00E-10 | 17.49317478 |
| 204162_at | NDC80 | 1.353121633 | 6.831606853 | 8.377413801 | 3.85E-15 | 1.08E-12 | 23.84760442 |
| 228956_at | UGT8 | -1.350808747 | 8.711132556 | -5.254219818 | 3.17E-07 | 3.87E-06 | 6.260450785 |
| 206190_at | GPR17 | -1.348561958 | 8.231770901 | -5.35543353 | 1.93E-07 | 2.50E-06 | 6.736772407 |
| 235591_at | SSTR1 | -1.345450127 | 5.6012358 | -8.049714467 | 3.31E-14 | 5.53E-12 | 21.75945572 |
| 213904_at | FRRS1L | -1.341700469 | 8.356876805 | -6.692236832 | 1.42E-10 | 5.36E-09 | 13.6657778 |
| 227062_at | MIR612///NEAT1 | 1.338055253 | 9.715895227 | 7.857786599 | 1.14E-13 | 1.50E-11 | 20.55898026 |
| 203240_at | FCGBP | 1.335264137 | 9.537433489 | 7.40827699 | 1.94E-12 | 1.50E-10 | 17.81662478 |
| 214710_s_at | CCNB1 | 1.334306796 | 7.199419351 | 8.934947557 | 8.91E-17 | 6.09E-14 | 27.50399293 |
| 223551_at | PKIB | 1.329212591 | 6.487856952 | 6.588859967 | 2.58E-10 | 8.88E-09 | 13.0902286 |
| 235417_at | SPOCD1 | 1.328241274 | 8.459206273 | 7.49920419 | 1.10E-12 | 9.54E-11 | 18.36325094 |
| 1552439_s_at | MEGF11 | -1.3260908 | 7.794974133 | -6.368096921 | 9.03E-10 | 2.57E-08 | 11.88231437 |
| 227662_at | SYNPO2 | -1.325698708 | 7.638357444 | -6.501559195 | 4.25E-10 | 1.36E-08 | 12.60907589 |
| 206026_s_at | TNFAIP6 | 1.319851487 | 6.515676205 | 7.905644813 | 8.40E-14 | 1.16E-11 | 20.85672031 |
| 205572_at | ANGPT2 | 1.3193953 | 7.63334842 | 7.231132128 | 5.75E-12 | 3.73E-10 | 16.76393168 |
| 202503_s_at | KIAA0101 | 1.317472933 | 8.754147344 | 8.183398869 | 1.38E-14 | 2.85E-12 | 22.60558069 |
| 213486_at | COPG2IT1 | -1.316288326 | 9.721750538 | -7.566871466 | 7.22E-13 | 6.78E-11 | 18.77275871 |
| 224646_x_at | H19///MIR675 | 1.311068717 | 6.690960268 | 5.154083255 | 5.16E-07 | 5.89E-06 | 5.796355049 |
| 230781_at | LINC01088 | -1.311062961 | 8.063801926 | -5.110981112 | 6.35E-07 | 7.03E-06 | 5.598806333 |
| 203324_s_at | CAV2 | 1.311006345 | 6.960186161 | 7.225818551 | 5.94E-12 | 3.83E-10 | 16.73260952 |
| 228547_at | NRXN1 | -1.308562149 | 9.41242258 | -6.448265273 | 5.75E-10 | 1.76E-08 | 12.31758036 |
| 231131_at | FAM133A | -1.306371985 | 7.050634827 | -10.48234736 | 1.45E-21 | 1.58E-17 | 38.22639203 |
| 226621_at | OSMR | 1.299801696 | 8.611630901 | 7.658691173 | 4.05E-13 | 4.27E-11 | 19.33207861 |
| 208659_at | CLIC1 | 1.295352001 | 9.648477497 | 9.073610291 | 3.43E-17 | 3.17E-14 | 28.43223699 |
| 219732_at | LPPR1 | -1.294606022 | 9.588937028 | -6.005553729 | 6.64E-09 | 1.36E-07 | 9.963469361 |
| 212793_at | DAAM2 | -1.28986213 | 9.686576287 | -6.870030501 | 5.02E-11 | 2.24E-09 | 14.67005282 |
| 228017_s_at | NKAIN4 | -1.289663168 | 9.219122225 | -6.13375316 | 3.31E-09 | 7.65E-08 | 10.63257594 |
| 228904_at | HOXB3 | 1.287823316 | 5.98067003 | 5.696512043 | 3.41E-08 | 5.58E-07 | 8.394250666 |
| 229778_at | SPX | -1.286579997 | 7.074035459 | -4.9495353 | 1.36E-06 | 1.37E-05 | 4.870844857 |
| 1554474_a_at | MOXD1 | 1.27733862 | 6.95700429 | 6.706296749 | 1.31E-10 | 5.01E-09 | 13.74453634 |
| 204159_at | CDKN2C | 1.275862013 | 9.316409206 | 8.135081833 | 1.90E-14 | 3.68E-12 | 22.29884285 |
| 203764_at | DLGAP5 | 1.269784168 | 6.181906565 | 7.816613185 | 1.49E-13 | 1.85E-11 | 20.30369418 |
| 205523_at | HAPLN1 | -1.263430231 | 6.549232568 | -6.336603022 | 1.08E-09 | 2.97E-08 | 11.71238914 |
| 202878_s_at | CD93 | 1.260695281 | 8.551061667 | 7.441699391 | 1.58E-12 | 1.28E-10 | 18.01706037 |
| 210222_s_at | RTN1 | -1.260664787 | 10.73185127 | -6.887882522 | 4.52E-11 | 2.06E-09 | 14.77188154 |
| 238663_x_at | GRIA4 | -1.255083208 | 7.496830569 | -7.585579302 | 6.42E-13 | 6.13E-11 | 18.88637869 |
| 1555564_a_at | CFI | 1.254212555 | 6.806271018 | 6.508743571 | 4.08E-10 | 1.31E-08 | 12.64850131 |
| 202870_s_at | CDC20 | 1.253923739 | 6.962161855 | 8.827279981 | 1.86E-16 | 1.04E-13 | 26.78825566 |
| 214930_at | SLITRK5 | -1.253711087 | 7.912140117 | -8.327085506 | 5.37E-15 | 1.38E-12 | 23.5238494 |
| 202627_s_at | SERPINE1 | 1.252771552 | 8.426529995 | 7.570300898 | 7.06E-13 | 6.68E-11 | 18.79357394 |
| 203146_s_at | GABBR1 | -1.25089286 | 10.47457607 | -9.267711743 | 8.89E-18 | 1.19E-14 | 29.74342707 |
| 235527_at | DLGAP1 | -1.250518584 | 7.537178629 | -6.508644414 | 4.08E-10 | 1.31E-08 | 12.64795696 |
| 201590_x_at | ANXA2 | 1.249914816 | 10.58555875 | 7.479856242 | 1.24E-12 | 1.05E-10 | 18.24658512 |
| 219787_s_at | ECT2 | 1.247732476 | 7.105992034 | 9.318810579 | 6.22E-18 | 8.83E-15 | 30.09083679 |
| 242907_at | GBP2 | 1.246667428 | 7.658104996 | 6.573671082 | 2.81E-10 | 9.56E-09 | 13.00619206 |
| 1555630_a_at | NARR///RAB34 | 1.245347204 | 8.567821483 | 7.760958845 | 2.12E-13 | 2.49E-11 | 19.95990707 |
| 202589_at | TYMS | 1.24263298 | 9.582393599 | 8.564734748 | 1.10E-15 | 4.46E-13 | 25.06202566 |
| 200916_at | TAGLN2 | 1.237425566 | 9.699613682 | 9.223916131 | 1.21E-17 | 1.50E-14 | 29.44640084 |
| 219368_at | NAP1L2 | -1.235259478 | 8.652579935 | -6.42881912 | 6.42E-10 | 1.93E-08 | 12.21164258 |
| 211959_at | IGFBP5 | 1.234267313 | 10.8798433 | 8.000071924 | 4.57E-14 | 6.99E-12 | 21.44731616 |
| 204723_at | SCN3B | -1.23378511 | 8.240959207 | -5.975488499 | 7.81E-09 | 1.57E-07 | 9.808071412 |
| 236538_at | GRIA2 | -1.23248961 | 9.649392316 | -6.309394512 | 1.25E-09 | 3.38E-08 | 11.56607407 |
| 212067_s_at | C1R | 1.231712506 | 9.229990671 | 7.23719825 | 5.54E-12 | 3.62E-10 | 16.79970817 |
| 201896_s_at | PSRC1 | 1.231639454 | 9.755514997 | 10.06456394 | 3.06E-20 | 2.09E-16 | 35.25884037 |
| 203213_at | CDK1 | 1.230783711 | 7.649303696 | 7.353044928 | 2.72E-12 | 2.01E-10 | 17.48665371 |
| 205173_x_at | CD58 | 1.230234344 | 7.774540705 | 8.415258988 | 2.99E-15 | 9.24E-13 | 24.09176998 |
| 200600_at | MSN | 1.229534366 | 10.13315263 | 8.928132587 | 9.33E-17 | 6.22E-14 | 27.45855792 |
| 209990_s_at | GABBR2 | -1.228107254 | 9.828682387 | -5.451410477 | 1.19E-07 | 1.65E-06 | 7.195083606 |
| 204825_at | MELK | 1.227060506 | 7.701385995 | 8.248533579 | 9.02E-15 | 2.05E-12 | 23.02072228 |
| 208690_s_at | PDLIM1 | 1.22496052 | 8.41019891 | 7.246215559 | 5.25E-12 | 3.48E-10 | 16.85292599 |
| 205290_s_at | BMP2 | -1.222676776 | 8.046377734 | -7.290588115 | 4.00E-12 | 2.77E-10 | 17.1154254 |
| 1555958_at | CRTAC1 | -1.222120477 | 7.533460001 | -8.902550582 | 1.11E-16 | 6.95E-14 | 27.28816179 |
| 204170_s_at | CKS2 | 1.222116254 | 8.579547299 | 7.984928987 | 5.04E-14 | 7.65E-12 | 21.35232637 |
| 204865_at | CA3 | 1.221734318 | 5.098242379 | 5.712602409 | 3.13E-08 | 5.20E-07 | 8.474396191 |
| 209098_s_at | JAG1 | 1.221312366 | 6.98419684 | 7.537108796 | 8.69E-13 | 8.00E-11 | 18.59235844 |
| 223500_at | CPLX1 | -1.217116989 | 7.760963623 | -5.742584452 | 2.68E-08 | 4.55E-07 | 8.624197259 |
| 213338_at | TMEM158 | 1.215721562 | 9.414595133 | 7.192806238 | 7.26E-12 | 4.49E-10 | 16.53834616 |
| 211719_x_at | FN1 | 1.212980881 | 11.723933 | 8.02182481 | 3.97E-14 | 6.25E-12 | 21.58395384 |
| 227949_at | PHACTR3 | -1.210648661 | 8.607804109 | -5.976336641 | 7.77E-09 | 1.56E-07 | 9.812447219 |
| 229201_at | PAK3 | -1.210366144 | 7.450013963 | -5.773455334 | 2.28E-08 | 3.94E-07 | 8.779064883 |
| 239293_at | NRSN1 | -1.209783228 | 7.916070259 | -8.036047199 | 3.62E-14 | 5.90E-12 | 21.673407 |
| 227013_at | LATS2 | 1.209599525 | 8.362070147 | 9.136830739 | 2.21E-17 | 2.24E-14 | 28.85781058 |
| 227425_at | REPS2 | -1.207505597 | 7.652258861 | -6.832164741 | 6.27E-11 | 2.72E-09 | 14.45465882 |
| 242344_at | GABRB2 | -1.204977689 | 7.191788116 | -3.846705548 | 0.000151803 | 0.00081275 | 0.430220959 |
| 203313_s_at | TGIF1 | 1.204838252 | 7.87891948 | 8.90123631 | 1.12E-16 | 6.95E-14 | 27.27941444 |
| 221024_s_at | SLC2A10 | 1.202153867 | 7.646555544 | 7.271504977 | 4.49E-12 | 3.04E-10 | 17.00240638 |
| 207828_s_at | CENPF | 1.200348601 | 8.545897895 | 8.613352627 | 7.94E-16 | 3.32E-13 | 25.37959821 |
| 226237_at | COL8A1 | 1.198220763 | 6.431875873 | 5.33842123 | 2.10E-07 | 2.69E-06 | 6.656206313 |
| 203755_at | BUB1B | 1.196251812 | 7.836137455 | 7.835720114 | 1.32E-13 | 1.70E-11 | 20.42206217 |
| 215506_s_at | DIRAS3 | 1.188819809 | 7.526813545 | 7.723203763 | 2.69E-13 | 3.03E-11 | 19.72753471 |
| 1553765_a_at | KLHL32 | -1.186902893 | 5.869073207 | -7.148504939 | 9.50E-12 | 5.57E-10 | 16.27856505 |
| 230560_at | STXBP6 | -1.185154815 | 7.087088101 | -5.051628418 | 8.43E-07 | 9.02E-06 | 5.328975415 |
| 205330_at | MN1 | -1.183290016 | 7.320237124 | -8.209365094 | 1.17E-14 | 2.50E-12 | 22.77085433 |
| 207659_s_at | MOBP | -1.182935435 | 8.589667815 | -4.180183222 | 4.02E-05 | 0.000259184 | 1.671778988 |
| 203373_at | SOCS2 | 1.177246198 | 8.418664825 | 6.676220913 | 1.56E-10 | 5.77E-09 | 13.57620188 |
| 235521_at | HOXA3 | 1.175374304 | 5.920563649 | 6.195580306 | 2.36E-09 | 5.74E-08 | 10.95898263 |
| 218585_s_at | DTL | 1.172261652 | 7.197462411 | 6.696253137 | 1.39E-10 | 5.26E-09 | 13.68826401 |
| 231223_at | CSMD1 | -1.169836555 | 7.152499571 | -7.507050162 | 1.05E-12 | 9.23E-11 | 18.41061511 |
| 230865_at | LIX1 | -1.169628089 | 8.632882399 | -5.38460996 | 1.67E-07 | 2.21E-06 | 6.8754169 |
| 203484_at | SEC61G | 1.168995504 | 10.6048917 | 7.792645565 | 1.73E-13 | 2.09E-11 | 20.15546001 |
| 227769_at | GPR27 | -1.167459007 | 8.780388542 | -6.113978966 | 3.69E-09 | 8.33E-08 | 10.52868868 |
| 236095_at | NTRK2 | -1.166083245 | 7.61254761 | -7.763008906 | 2.09E-13 | 2.46E-11 | 19.97254433 |
| 213920_at | CUX2 | -1.165077133 | 6.371254942 | -6.288864026 | 1.41E-09 | 3.72E-08 | 11.45597126 |
| 227249_at | NDE1 | 1.164609773 | 7.574731698 | 10.51666285 | 1.12E-21 | 1.53E-17 | 38.47221383 |
| 218009_s_at | PRC1 | 1.162083299 | 8.010822228 | 8.327974993 | 5.34E-15 | 1.38E-12 | 23.5295619 |
| 232833_at | RP6-201G10.2 | -1.161999209 | 7.513930091 | -7.301167816 | 3.75E-12 | 2.63E-10 | 17.17816545 |
| 202022_at | ALDOC | -1.159490231 | 11.33555772 | -7.506145728 | 1.05E-12 | 9.26E-11 | 18.40515368 |
| 201110_s_at | THBS1 | 1.158857463 | 5.6092781 | 5.145262903 | 5.38E-07 | 6.10E-06 | 5.755820102 |
| 1558706_a_at | ATOH8 | -1.156210531 | 6.305616749 | -7.294715326 | 3.90E-12 | 2.71E-10 | 17.13989373 |
| 210292_s_at | PCDH11X///PCDH11Y | -1.153689973 | 6.801102494 | -6.287279843 | 1.42E-09 | 3.74E-08 | 11.44748623 |
| 210052_s_at | TPX2 | 1.152806307 | 7.65916383 | 8.448859214 | 2.39E-15 | 7.73E-13 | 24.30905682 |
| 214762_at | ATP6V1G2 | -1.152530304 | 9.366550796 | -7.612564169 | 5.42E-13 | 5.32E-11 | 19.05057462 |
| 201289_at | CYR61 | 1.151089829 | 9.160408951 | 6.144808484 | 3.12E-09 | 7.27E-08 | 10.69076452 |
| 213993_at | SPON1 | -1.148942547 | 7.971877159 | -6.327139634 | 1.14E-09 | 3.10E-08 | 11.66144787 |
| 205110_s_at | FGF13 | -1.148581057 | 8.15845065 | -5.487195415 | 9.95E-08 | 1.41E-06 | 7.367601103 |
| 228608_at | NALCN | -1.146859746 | 8.800659148 | -7.066144531 | 1.56E-11 | 8.47E-10 | 15.79841328 |
| 204844_at | ENPEP | 1.146556065 | 5.853280055 | 7.408616826 | 1.93E-12 | 1.50E-10 | 17.81865991 |
| 238047_at | ARHGAP36 | -1.145876294 | 6.027236083 | -6.235521181 | 1.89E-09 | 4.78E-08 | 11.171115 |
| 231358_at | MRO | -1.140496427 | 8.577622106 | -7.056944904 | 1.65E-11 | 8.87E-10 | 15.74500885 |
| 211006_s_at | KCNB1 | -1.140377494 | 8.134639878 | -7.43197678 | 1.67E-12 | 1.35E-10 | 17.95869453 |
| 225612_s_at | B3GNT5 | 1.138781952 | 8.218458066 | 7.47243652 | 1.30E-12 | 1.09E-10 | 18.20189537 |
| 231798_at | NOG | -1.138582632 | 6.178324635 | -8.651019464 | 6.16E-16 | 2.83E-13 | 25.62629938 |
| 207957_s_at | PRKCB | -1.137324109 | 8.368243688 | -4.856161024 | 2.11E-06 | 2.00E-05 | 4.458559501 |
| 227628_at | GPX8 | 1.13462978 | 6.150879859 | 7.238682091 | 5.49E-12 | 3.60E-10 | 16.80846247 |
| 225081_s_at | CDCA7L | 1.133736892 | 8.585236227 | 8.042896969 | 3.46E-14 | 5.70E-12 | 21.7165223 |
| 207714_s_at | SERPINH1 | 1.132234875 | 8.603611141 | 8.485384813 | 1.87E-15 | 6.48E-13 | 24.54580007 |
| 229461_x_at | NEGR1 | -1.130694 | 7.183697891 | -5.460628647 | 1.14E-07 | 1.59E-06 | 7.239439357 |
| 214608_s_at | EYA1 | -1.130062605 | 7.734789335 | -7.089213447 | 1.36E-11 | 7.52E-10 | 15.93253229 |
| 200986_at | SERPING1 | 1.128672265 | 8.766162092 | 6.766533175 | 9.22E-11 | 3.74E-09 | 14.08324806 |
| 229400_at | HOXD10 | 1.127553462 | 6.900868173 | 7.109779259 | 1.20E-11 | 6.80E-10 | 16.05234201 |
| 206858_s_at | HOXC6 | 1.126016076 | 6.848743901 | 6.696794172 | 1.38E-10 | 5.25E-09 | 13.69129383 |
| 201645_at | TNC | 1.125264716 | 10.23098841 | 6.403075732 | 7.42E-10 | 2.18E-08 | 12.07174999 |
| 229151_at | SLC14A1 | -1.125021787 | 7.284493239 | -4.200040072 | 3.71E-05 | 0.000241825 | 1.748546174 |
| 204822_at | TTK | 1.124977843 | 5.995543167 | 7.301086524 | 3.75E-12 | 2.63E-10 | 17.17768314 |
| 200771_at | LAMC1 | 1.12446162 | 9.307180212 | 9.389514357 | 3.78E-18 | 6.47E-15 | 30.5730306 |
| 231980_at | DOK6 | -1.123656856 | 6.263316244 | -6.264888774 | 1.61E-09 | 4.16E-08 | 11.32772325 |
| 230923_at | FAM19A1 | -1.12302411 | 6.366843333 | -5.927963076 | 1.01E-08 | 1.94E-07 | 9.563616943 |
| 238521_at | FGF12 | -1.122545775 | 8.20147398 | -6.697231634 | 1.38E-10 | 5.24E-09 | 13.69374377 |
| 211564_s_at | PDLIM4 | 1.122337004 | 6.40435041 | 5.942762041 | 9.30E-09 | 1.81E-07 | 9.639581003 |
| 238081_at | WDFY3-AS2 | -1.119726228 | 7.202949858 | -8.245765642 | 9.18E-15 | 2.07E-12 | 23.00304262 |
| 213258_at | TFPI | 1.118930252 | 7.437590457 | 5.301440213 | 2.52E-07 | 3.16E-06 | 6.481775154 |
| 209072_at | MBP | -1.116929501 | 11.65092822 | -3.842498994 | 0.000154281 | 0.000823659 | 0.415141072 |
| 233002_at | PPP4R4 | -1.116222291 | 5.504254768 | -6.656367867 | 1.75E-10 | 6.40E-09 | 13.46537184 |
| 230913_at | ABCG1 | -1.115469364 | 7.929331536 | -9.043463418 | 4.22E-17 | 3.55E-14 | 28.22981703 |
| 202672_s_at | ATF3 | 1.11405492 | 8.744483983 | 8.095018539 | 2.47E-14 | 4.35E-12 | 22.0452961 |
| 235342_at | SPOCK3 | -1.113406295 | 7.624993422 | -4.194816728 | 3.79E-05 | 0.000246267 | 1.72832199 |
| 213418_at | HSPA6 | 1.112897618 | 7.359820327 | 6.461004467 | 5.35E-10 | 1.66E-08 | 12.38710363 |
| 227845_s_at | SHD | -1.112132821 | 8.035562453 | -6.907300482 | 4.03E-11 | 1.87E-09 | 14.88284538 |
| 200660_at | S100A11 | 1.111203402 | 8.382597796 | 7.346810142 | 2.83E-12 | 2.08E-10 | 17.44950446 |
| 202430_s_at | PLSCR1 | 1.110758214 | 7.399960998 | 7.822803048 | 1.43E-13 | 1.80E-11 | 20.34202156 |
| 203963_at | CA12 | 1.107154363 | 8.750753056 | 6.033219384 | 5.72E-09 | 1.20E-07 | 10.10697743 |
| 202207_at | ARL4C | 1.104771563 | 9.73286021 | 7.481996553 | 1.23E-12 | 1.04E-10 | 18.25948162 |
| 211276_at | TCEAL2 | -1.104240549 | 10.09859081 | -6.310172756 | 1.25E-09 | 3.37E-08 | 11.5702528 |
| 228108_at | PPM1L | -1.102937674 | 8.54628515 | -8.294692871 | 6.65E-15 | 1.63E-12 | 23.31604899 |
| 237696_at | RP11-231C18.1 | -1.102447913 | 7.027483563 | -7.133859071 | 1.04E-11 | 5.99E-10 | 16.19291362 |
| 209803_s_at | PHLDA2 | 1.102121166 | 5.991151963 | 5.794353957 | 2.04E-08 | 3.59E-07 | 8.884264375 |
| 238877_at | EYA4 | 1.101880306 | 6.561053037 | 5.72403189 | 2.95E-08 | 4.94E-07 | 8.531431216 |
| 231372_at | SLC25A48 | -1.10087012 | 7.000961261 | -7.615253246 | 5.33E-13 | 5.25E-11 | 19.06695672 |
| 205737_at | KCNQ2 | -1.099474112 | 8.201816359 | -6.449386195 | 5.71E-10 | 1.75E-08 | 12.32369381 |
| 206899_at | NTSR2 | -1.098924858 | 7.769715101 | -6.312959966 | 1.23E-09 | 3.33E-08 | 11.58522162 |
| 236641_at | KIF14 | 1.09799307 | 5.558320908 | 7.651214223 | 4.25E-13 | 4.39E-11 | 19.28637715 |
| 201438_at | COL6A3 | 1.095738974 | 7.36982423 | 5.190847246 | 4.32E-07 | 5.05E-06 | 5.965909837 |
| 227984_at | LMF1 | -1.093506628 | 10.03215842 | -6.12445604 | 3.48E-09 | 7.96E-08 | 10.58370112 |
| 206013_s_at | ACTL6B | -1.093444203 | 6.546663682 | -7.208434828 | 6.60E-12 | 4.17E-10 | 16.63024156 |
| 218983_at | C1RL | 1.091941802 | 6.718693157 | 7.126623951 | 1.08E-11 | 6.21E-10 | 16.15064404 |
| 220116_at | KCNN2 | -1.089857927 | 8.369597608 | -6.230942666 | 1.94E-09 | 4.89E-08 | 11.1467474 |
| 202508_s_at | SNAP25 | -1.087319558 | 10.63985204 | -4.232988652 | 3.24E-05 | 0.000214792 | 1.876621505 |
| 234996_at | CALCRL | -1.086487609 | 6.718707615 | -6.527938245 | 3.66E-10 | 1.19E-08 | 12.75398592 |
| 205547_s_at | TAGLN | 1.0863013 | 8.723335845 | 5.816779002 | 1.82E-08 | 3.25E-07 | 8.99746881 |
| 206280_at | CDH18 | -1.08514983 | 7.175376523 | -6.540061042 | 3.41E-10 | 1.13E-08 | 12.82071972 |
| 218755_at | KIF20A | 1.081022272 | 6.770788727 | 8.102987455 | 2.34E-14 | 4.24E-12 | 22.09567106 |
| 223276_at | SMIM3 | 1.080145381 | 8.505109465 | 7.206316819 | 6.69E-12 | 4.21E-10 | 16.61778015 |
| 1557256_a_at | GABRB1 | -1.080117175 | 7.504287962 | -5.418292344 | 1.41E-07 | 1.91E-06 | 7.036212515 |
| 214954_at | SUSD5 | -1.07983565 | 5.724486359 | -5.245208279 | 3.31E-07 | 4.02E-06 | 6.218392836 |
| 225647_s_at | CTSC | 1.07920299 | 6.115950462 | 5.99922004 | 6.87E-09 | 1.41E-07 | 9.93068413 |
| 205651_x_at | RAPGEF4 | -1.078894336 | 8.364518925 | -6.208067254 | 2.20E-09 | 5.43E-08 | 11.02519602 |
| 218807_at | VAV3 | 1.07863934 | 7.865841628 | 7.707909978 | 2.97E-13 | 3.31E-11 | 19.63360193 |
| 226913_s_at | SOX8 | -1.076149678 | 10.99962162 | -5.526502076 | 8.16E-08 | 1.19E-06 | 7.558113528 |
| 235489_at | RHOJ | 1.073076127 | 7.9571744 | 6.962582044 | 2.90E-11 | 1.42E-09 | 15.1999036 |
| 237094_at | FAM19A5 | -1.071640287 | 8.19903315 | -6.8386247 | 6.04E-11 | 2.63E-09 | 14.4913481 |
| 221766_s_at | FAM46A | 1.069515951 | 8.545732213 | 7.400968988 | 2.03E-12 | 1.57E-10 | 17.77287463 |
| 209839_at | DNM3 | -1.067730801 | 8.928647128 | -6.016020971 | 6.28E-09 | 1.31E-07 | 10.01770765 |
| 214376_at | MAPK10 | -1.067395833 | 8.403720215 | -8.050385731 | 3.30E-14 | 5.53E-12 | 21.76368417 |
| 227812_at | TNFRSF19 | 1.066373958 | 7.766840868 | 6.174000045 | 2.65E-09 | 6.34E-08 | 10.84478068 |
| 238603_at | LINC00925 | -1.066238137 | 8.180558898 | -7.982451611 | 5.12E-14 | 7.72E-12 | 21.33679616 |
| 230497_at | CELF5 | -1.066013557 | 8.35162764 | -6.191625922 | 2.41E-09 | 5.84E-08 | 10.93803438 |
| 212558_at | SPRY1 | 1.065785222 | 8.740185902 | 7.512210123 | 1.02E-12 | 9.01E-11 | 18.44178143 |
| 208949_s_at | LGALS3 | 1.065702548 | 10.6979166 | 6.071155397 | 4.66E-09 | 1.01E-07 | 10.30455559 |
| 210246_s_at | ABCC8 | -1.065642207 | 8.086147103 | -9.053656967 | 3.93E-17 | 3.36E-14 | 28.29822367 |
| 219331_s_at | KLHDC8A | 1.065568465 | 8.366140699 | 7.362592654 | 2.57E-12 | 1.91E-10 | 17.54358179 |
| 219890_at | CLEC5A | 1.065487928 | 5.763455588 | 6.481139386 | 4.77E-10 | 1.50E-08 | 12.4971871 |
| 218542_at | CEP55 | 1.065459172 | 6.220436771 | 7.860966604 | 1.12E-13 | 1.48E-11 | 20.57873054 |
| 225314_at | OCIAD2 | 1.064886254 | 8.795550897 | 6.147750964 | 3.07E-09 | 7.17E-08 | 10.70626492 |
| 1559072_a_at | ELFN2 | -1.063627724 | 8.765512741 | -8.493952004 | 1.77E-15 | 6.24E-13 | 24.60140971 |
| 202917_s_at | S100A8 | 1.062629785 | 7.955449752 | 4.510770541 | 9.92E-06 | 7.65E-05 | 2.990495187 |
| 241399_at | FAM19A2 | -1.061486866 | 6.947545363 | -6.513855654 | 3.96E-10 | 1.28E-08 | 12.67657342 |
| 226623_at | PHYHIPL | -1.061249989 | 10.61450443 | -8.220889312 | 1.08E-14 | 2.36E-12 | 22.84430082 |
| 235066_at | MAP4 | -1.059083091 | 7.531999713 | -4.068699398 | 6.33E-05 | 0.000383505 | 1.246661158 |
| 221489_s_at | SPRY4 | 1.057639677 | 8.867988563 | 6.615635828 | 2.21E-10 | 7.81E-09 | 13.23870351 |
| 213664_at | SLC1A1 | -1.056270148 | 8.681405828 | -6.239959813 | 1.85E-09 | 4.69E-08 | 11.19475054 |
| 204517_at | PPIC | 1.055621828 | 8.159840932 | 7.105046027 | 1.24E-11 | 6.97E-10 | 16.02474747 |
| 206196_s_at | RUNDC3A | -1.054773675 | 8.7045424 | -6.132724953 | 3.33E-09 | 7.68E-08 | 10.62716799 |
| 201617_x_at | CALD1 | 1.052877284 | 8.135767156 | 7.879053731 | 9.96E-14 | 1.34E-11 | 20.6911563 |
| 203570_at | LOXL1 | 1.052288414 | 6.877630923 | 6.361574548 | 9.37E-10 | 2.65E-08 | 11.84707325 |
| 208892_s_at | DUSP6 | 1.052288356 | 9.127893409 | 6.472689265 | 5.01E-10 | 1.56E-08 | 12.45095824 |
| 215870_s_at | PLA2G5 | 1.05196367 | 7.356910009 | 5.091508415 | 6.97E-07 | 7.66E-06 | 5.509997538 |
| 214111_at | OPCML | -1.051212212 | 8.937590934 | -5.091506067 | 6.97E-07 | 7.66E-06 | 5.509986843 |
| 214279_s_at | NDRG2 | -1.050738513 | 9.380160632 | -7.50710193 | 1.05E-12 | 9.23E-11 | 18.41092773 |
| 209835_x_at | CD44 | 1.048090034 | 8.345955196 | 6.914797927 | 3.85E-11 | 1.80E-09 | 14.92574593 |
| 222379_at | KCNE4 | 1.04736556 | 6.825023747 | 6.431033335 | 6.34E-10 | 1.91E-08 | 12.22369358 |
| 212298_at | NRP1 | 1.046911084 | 7.402154577 | 6.562558247 | 3.00E-10 | 1.01E-08 | 12.94479353 |
| 236761_at | LHFPL3 | -1.046817897 | 9.744107453 | -4.140115593 | 4.74E-05 | 0.00029868 | 1.51783857 |
| 201506_at | TGFBI | 1.046641125 | 9.451264927 | 5.784264669 | 2.15E-08 | 3.76E-07 | 8.833440802 |
| 210721_s_at | PAK7 | -1.046237427 | 5.730960582 | -6.430950959 | 6.34E-10 | 1.91E-08 | 12.22324519 |
| 221623_at | BCAN | -1.043766261 | 7.828509862 | -6.991897749 | 2.44E-11 | 1.24E-09 | 15.36872686 |
| 208782_at | FSTL1 | 1.04367431 | 10.27532683 | 8.384162549 | 3.68E-15 | 1.05E-12 | 23.89110063 |
| 202705_at | CCNB2 | 1.042570692 | 7.465358794 | 7.96410726 | 5.76E-14 | 8.45E-12 | 21.22188702 |
| 230496_at | AMER2 | -1.039255734 | 10.87141998 | -5.850765296 | 1.52E-08 | 2.78E-07 | 9.169667553 |
| 209642_at | BUB1 | 1.038860038 | 5.869713708 | 7.691145312 | 3.30E-13 | 3.62E-11 | 19.53076578 |
| 207103_at | KCND2 | -1.036171213 | 8.127449266 | -4.900219862 | 1.72E-06 | 1.68E-05 | 4.652292375 |
| 228010_at | PPP2R2C | -1.034171158 | 8.777690953 | -4.895421708 | 1.76E-06 | 1.71E-05 | 4.631124296 |
| 218663_at | NCAPG | 1.033661844 | 5.321683864 | 7.688787198 | 3.35E-13 | 3.66E-11 | 19.51631185 |
| 213222_at | PLCB1 | -1.033241834 | 9.876959191 | -6.968778056 | 2.79E-11 | 1.38E-09 | 15.23554559 |
| 209875_s_at | SPP1 | 1.030724655 | 12.20317782 | 7.530323418 | 9.07E-13 | 8.24E-11 | 18.55129236 |
| 1569348_at | LOC102725451///TPTEP1 | -1.027746082 | 5.716457123 | -6.53981672 | 3.42E-10 | 1.13E-08 | 12.81937391 |
| 223315_at | NTN4 | -1.027547659 | 5.993201736 | -7.501836432 | 1.08E-12 | 9.45E-11 | 18.37913766 |
| 200791_s_at | IQGAP1 | 1.027480772 | 9.675169761 | 9.567873011 | 1.07E-18 | 2.52E-15 | 31.79695098 |
| 231771_at | GJB6 | -1.027414883 | 6.688629946 | -3.925063482 | 0.000111985 | 0.000626887 | 0.713791875 |
| 202863_at | SP100 | 1.024334888 | 6.941353043 | 7.826845118 | 1.39E-13 | 1.77E-11 | 20.36705972 |
| 204753_s_at | HLF | -1.024302558 | 7.589475023 | -5.948641093 | 9.01E-09 | 1.77E-07 | 9.669797953 |
| 208161_s_at | ABCC3 | 1.02422303 | 7.605991046 | 6.52753907 | 3.66E-10 | 1.20E-08 | 12.75179001 |
| 202095_s_at | BIRC5 | 1.024042188 | 7.351026368 | 7.274521747 | 4.41E-12 | 3.00E-10 | 17.02026035 |
| 1566968_at | SPRY4-IT1 | 1.023995402 | 7.543210926 | 6.897100002 | 4.28E-11 | 1.96E-09 | 14.82452846 |
| 203474_at | IQGAP2 | 1.023936941 | 7.192592102 | 7.546310815 | 8.21E-13 | 7.59E-11 | 18.64808725 |
| 201105_at | LGALS1 | 1.021703386 | 11.29378263 | 7.434717729 | 1.64E-12 | 1.33E-10 | 17.97514384 |
| 213106_at | ATP8A1 | -1.021636472 | 9.339495173 | -5.993127697 | 7.10E-09 | 1.45E-07 | 9.899172504 |
| 236448_at | UNC5A | -1.021202541 | 8.143484665 | -6.199464546 | 2.31E-09 | 5.64E-08 | 10.9795688 |
| 212671_s_at | HLA-DQA1///HLA-DQA2///LOC100509457 | 1.020660567 | 8.302772078 | 4.917525528 | 1.58E-06 | 1.56E-05 | 4.728781644 |
| 209183_s_at | C10orf10 | 1.01996505 | 8.919406051 | 7.251054226 | 5.09E-12 | 3.39E-10 | 16.88150025 |
| 226722_at | FAM20C | 1.019291196 | 9.032056651 | 8.493417885 | 1.78E-15 | 6.24E-13 | 24.59794184 |
| 222150_s_at | GSAP | 1.018269608 | 7.861247598 | 7.438263926 | 1.61E-12 | 1.31E-10 | 17.9964314 |
| 202580_x_at | FOXM1 | 1.01814477 | 6.857814697 | 8.060937512 | 3.08E-14 | 5.23E-12 | 21.83017932 |
| 241365_at | SATB1 | -1.017739997 | 6.862054429 | -7.492560881 | 1.15E-12 | 9.86E-11 | 18.32317124 |
| 213905_x_at | BGN | 1.01703982 | 8.338811227 | 7.368720624 | 2.47E-12 | 1.85E-10 | 17.58014451 |
| 228771_at | ADRBK2 | -1.015454213 | 8.321632388 | -7.555351017 | 7.76E-13 | 7.21E-11 | 18.70287735 |
| 229300_at | RAB3C | -1.015271233 | 7.017648726 | -5.347486272 | 2.00E-07 | 2.59E-06 | 6.699110861 |
| 205499_at | SRPX2 | 1.015100272 | 6.601628581 | 6.32224014 | 1.17E-09 | 3.18E-08 | 11.63509551 |
| 230287_at | SGSM1 | -1.014215832 | 7.680781063 | -8.412311508 | 3.05E-15 | 9.26E-13 | 24.07273191 |
| 200650_s_at | LDHA | 1.012890863 | 11.95115498 | 8.894179178 | 1.18E-16 | 7.00E-14 | 27.2324559 |
| 229057_at | SCN2A | -1.012799113 | 7.878053822 | -4.715777722 | 3.99E-06 | 3.49E-05 | 3.850951918 |
| 213746_s_at | FLNA | 1.012251717 | 8.278320918 | 8.288866306 | 6.91E-15 | 1.67E-12 | 23.27871951 |
| 207093_s_at | LOC101927057///OMG | -1.011386343 | 10.25829249 | -6.314100448 | 1.22E-09 | 3.31E-08 | 11.591348 |
| 229875_at | ZDHHC22 | -1.011002027 | 8.71191778 | -8.733227559 | 3.53E-16 | 1.87E-13 | 26.16670663 |
| 209755_at | NMNAT2 | -1.010737305 | 8.56882507 | -6.48377921 | 4.70E-10 | 1.49E-08 | 12.51163779 |
| 202833_s_at | SERPINA1 | 1.010506171 | 7.154245257 | 6.311120579 | 1.24E-09 | 3.36E-08 | 11.57534259 |
| 218355_at | KIF4A | 1.009999912 | 7.179768611 | 7.479064905 | 1.25E-12 | 1.06E-10 | 18.24181749 |
| 204962_s_at | CENPA///SLC35F6 | 1.008620257 | 6.67999085 | 7.739864573 | 2.42E-13 | 2.77E-11 | 19.82999263 |
| 211184_s_at | USH1C | -1.008021221 | 7.025322637 | -8.206930593 | 1.19E-14 | 2.50E-12 | 22.75534619 |
| 204529_s_at | TOX | -1.007874812 | 7.897144777 | -7.073093992 | 1.50E-11 | 8.18E-10 | 15.8387859 |
| 232122_s_at | VEPH1 | -1.007742736 | 6.384638041 | -6.319055579 | 1.19E-09 | 3.23E-08 | 11.61797496 |
| 232059_at | DSCAML1 | -1.007722419 | 7.380910074 | -8.608693922 | 8.19E-16 | 3.39E-13 | 25.34912572 |
| 202990_at | PYGL | 1.00758936 | 8.001284166 | 7.608611821 | 5.55E-13 | 5.43E-11 | 19.02650305 |
| 212294_at | GNG12 | 1.0073549 | 9.535411888 | 8.361027075 | 4.29E-15 | 1.18E-12 | 23.74207164 |
| 223550_s_at | CA10 | -1.006187083 | 7.74293604 | -5.368600803 | 1.80E-07 | 2.36E-06 | 6.799268682 |
| 36711_at | MAFF | 1.005905233 | 8.003401891 | 5.797303566 | 2.01E-08 | 3.54E-07 | 8.899135396 |
| 213844_at | HOXA5 | 1.005428147 | 5.97931816 | 6.995708945 | 2.38E-11 | 1.21E-09 | 15.39070963 |
| 202998_s_at | LOXL2 | 1.005261761 | 7.947222928 | 7.331538069 | 3.11E-12 | 2.23E-10 | 17.35859279 |
| 203725_at | GADD45A | 1.004792248 | 9.671765426 | 7.58787425 | 6.33E-13 | 6.06E-11 | 18.9003288 |
| 219671_at | HPCAL4 | -1.004335649 | 7.655689697 | -5.185867299 | 4.42E-07 | 5.15E-06 | 5.942885699 |
| 235940_at | C9orf64 | 1.003824911 | 6.950039596 | 7.026543446 | 1.98E-11 | 1.03E-09 | 15.56885509 |
| 228882_at | TUB | -1.003623428 | 9.012597809 | -8.971200164 | 6.94E-17 | 5.13E-14 | 27.74598233 |
| 213436_at | CNR1 | 1.002647551 | 8.755100785 | 5.385956681 | 1.65E-07 | 2.19E-06 | 6.881830801 |
| 206584_at | LY96 | 1.002494791 | 7.53441877 | 5.697215365 | 3.39E-08 | 5.57E-07 | 8.397750261 |
| 203798_s_at | VSNL1 | -1.001276929 | 7.089544079 | -3.307550421 | 0.001078672 | 0.004431316 | -1.381365078 |
| 205528_s_at | RUNX1T1 | -1.00101707 | 6.444032943 | -9.498764108 | 1.75E-18 | 3.55E-15 | 31.32145511 |
| DEGs: differentially expressed genes; HGG: high-grade glioma; LGG: low-grade glioma; log2FC: log2FoldChange. | | | | | | | |
